# Development of the basic architecture of neocortical circuitry in the human fetus as revealed by the coupling spatiotemporal pattern of synaptogenesis along with microstructure and macroscale in vivo MR imaging

**DOI:** 10.1007/s00429-024-02838-9

**Published:** 2024-08-05

**Authors:** Ivica Kostović

**Affiliations:** Croatian Institute for Brain Research, School of Medicine, University of Zagreb, Zagreb, Croatia

**Keywords:** Human fetal cortex, Synaptogenesis, Transient compartments, In vivo MR imaging

## Abstract

In humans, a quantifiable number of cortical synapses appears early in fetal life. In this paper, we present a bridge across different scales of resolution and the distribution of synapses across the transient cytoarchitectonic compartments: marginal zone (MZ), cortical plate (CP), subplate (SP), and in vivo MR images. The tissue of somatosensory cortex (7–26 postconceptional weeks (PCW)) was prepared for electron microscopy, and classified synapses with a determined subpial depth were used for creating histograms matched to the histological sections immunoreacted for synaptic markers and aligned to in vivo MR images (1.5 T) of corresponding fetal ages (maternal indication). Two time periods and laminar patterns of synaptogenesis were identified: an early and midfetal two-compartmental distribution (MZ and SP) and a late fetal three-compartmental distribution (CP synaptogenesis). During both periods, a voluminous, synapse-rich SP was visualized on the in vivo MR. Another novel finding concerns the phase of secondary expansion of the SP (13 PCW), where a quantifiable number of synapses appears in the upper SP. This lamina shows a T2 intermediate signal intensity below the low signal CP. In conclusion, the early fetal appearance of synapses shows early differentiation of putative genetic mechanisms underlying the synthesis, transport and assembly of synaptic proteins. “Pioneering” synapses are likely to play a morphogenetic role in constructing of fundamental circuitry architecture due to interaction between neurons. They underlie spontaneous, evoked, and resting state activity prior to ex utero experience. Synapses can also mediate genetic and environmental triggers, adversely altering the development of cortical circuitry and leading to neurodevelopmental disorders.

## Introduction

The number of cortical synapses changes throughout the life of a person, where the initial synaptogenesis starts during prenatal life (Molliver et al. 1973); an explosion in the number of synapses occurs postnatally, peaking between 1 and 2.5 years of age (Huttenlocher and Dabholkar 1997; Petanjek et al. 2011), which is followed by an initial pruning of spine synapses and a developmental plateau during late childhood and early adolescence (Huttenlocher and Dabholkar 1997; Petanjek et al. 2011), thus achieving the adult number after the second pruning in late adolescence (Petanjek et al. 2011).

Nevertheless, very few papers are dealing with direct ultrastructural analysis of exact timing of the appearance, spatial distribution and morphological features of synapses across the entire thickness of the fetal cortex (Molliver et al. 1973; Kostovic and Molliver 1974; Kostovic and Rakic 1990) or individual cortical layers (Molliver et al. 1973; Zecevic 1998).

In the study of compartmental distribution of synapses in the human fetal cerebrum it is essential to determine and relate: (1) laminar distribution of synapses to other neurogenetic events (Bystron et al. 2008; Kostović et al. 2019b; Kostović 2020); (2) explore their histological correlates (microscale) with in vivo, in utero images (macroscale) and (3) evaluate ultrastructural features of early synapses. The first objective can be achieved only by precise correlation of the EM laminar pattern of synaptogenesis with spatiotemporal laminar cytoarchitectonic development on the postmortem human material (Molliver et al. 1973). The second objective seems to be more demanding due to the ethical limitations of human in vivo neuroimaging. However, what is encouraging is the visualization of the voluminous transient cytoarchitectonically defined subplate compartment, which is synapse-rich (Kostović 2020), through magnetic resonance using structural and diffusion tension in vivo and in vitro imaging (Lan et al. 2000; Maas et al. 2004; Judas et al. 2005; Radoš et al. 2006; Prayer et al. 2006; Bayatti et al. 2008; Perkins et al. 2008; Huang et al. 2009; Dudink et al. 2010; Widjaja et al. 2010; Corbett-Detig et al. 2011; Kostović et al. 2014; Vasung et al. 2016, 2017; Miloš et al. 2020; Wang et al. 2023; Calixto et al. 2023). In vitro and in vivo MR images of the developing cortical laminae can be reliably transposed to the histochemically and immunocytochemically defined compartments (Kostović et al. 2014, 2019a). The precise correlation with annotations on the cellular resolution of the anatomical and molecular atlases of the prenatal human brain (Ding et al. 2022) seems to be less precise, as discussed by Clowry 2024.

Based on these developmental questions and our continuing interest in the development of the human cortex (Molliver et al. 1973; Kostovic and Rakic 1990; Kostović et al. 2014, 2019b; Kostović 2020), we have defined the major objective of this study to be the bridging of extreme scales in analysis of the fetal synaptogenesis by spanning from the laminar distribution of electromicroscopically identified synapses, matched to the transient cytoarchitectonic cellular compartments visualized on the immunoreacted histological sections to the macroscale of in vivo conventional MR images of the human fetal cortex during the critical period of initial synaptogenesis between 8 and 26 postconceptional weeks (PCW).

The specific aims were: (1) To analyze the distribution (spatial pattern) of synapses at different cortical (subpial) depths and developmental periods (temporal pattern) using systematic electromicroscopic vertical probes throughout the entire thickness of the cerebral wall in the region of the prospective somatosensory cortex on post mortem human fetal (fixed) material and compare histograms of synaptic distribution with the landmarks of transient compartments (CP, MZ, SP) on the adjacent Nissl-stained sections. (2) To estimate the percentage of each classified type of synapse within the different synapse-rich compartments (CP, MZ, SP) and ages. (3) To correlate histograms of the laminar distribution of EM-identified synapses with a laminar expression for immunocytochemical synaptic, fibrillar and extracellular matrix markers at the light microscopic level. (4) To compare the laminar delineation of synapse-rich compartments with the laminar pattern of MR signal intensity within the cerebral wall on in vivo, in utero images of the fetal cerebrum obtained from diagnostic (mother’s indication) MR examination.

We endeavored to harvest new data on the developmental appearance and spatiotemporal distribution of synapse-rich compartments in a specific cortical region, which can be used for future studies into functional, behavioral and molecular development of a normal cortex. In addition, these new multiscale spatiotemporal data are normative for researching alterations of synapse formation due to the different antenatal triggers, which in turn lead to neurodevelopmental disorders and make up the largest category of disorders in the pediatric population (Trauner 2019).

## Material and methods

The material used in this study originated from three sources: (1) Postmortem EM embedded blocks of the prospective somatosensory cortex (age range 7.5–26 PCW), cut ultrathin and then analyzed using transmission EM to determine the spatial distribution and ultrastructural features of synapses and ultrastructural features of the neuropil; (2) Human postmortal cerebral tissue treated using histochemistry and immunocytochemistry (age range 10.5–26 PCW); (3) In vivo, in utero MR images of fetuses without detectable abnormalities (age range 10.5–29 PCW). The list of all specimens used as these three sources is presented in Table 1. Postmortem tissue specimens (from first and second sources) come from the Zagreb Neuroembryological Collection, which contains specimens collected since 1974 and supplemented with in vitro and in vivo imaging data after 2000. All specimens and in vivo data were collected with IRB approval monitoring by the Ethics Committee at the School of Medicine of the University of Zagreb while also adhering to the rules of the Helsinki Declaration. In each case, parental consent for postmortem examination was obtained. For in vivo imaging cases, additional approval was obtained from the Ethics Committee of the University Hospital Centre Zagreb with the informed consent of patients Table 1.

**Table 1:** List of specimens

EM specimens				MRI specimens				IHC (paraffin sections)				AChE histochemistry (frozen sections			
Specimen	CRL (mm)	Age (PCW)	Cause of death	Specimen	CRL (mm)	Age (PCW)	Diagnosis	Specimen	CRL (mm)	Age (PCW)	Cause of death	Specimen	CRL (mm)	Age (PCW)	CRL (mm)
EM1	24	7.5	m.a	IvMI 1	–	11	*Tumor ovarii*	IHC1	–	10.5	m.a	AChE1	–	10	m.a
EM2	28	8	m.a	IvMI 2	–	10.5	o.m.i	IHC2	–	13	m.a	AChE2	–	15	s.a
EM3	37	8.5	m.a	IvMI 3	–	13	*Myoma uteri*	IHC3	–	17	s.a	AChE3	–	17	s.a
EM4	45	9	m.a	IvMI 4	–	14	o.m.i	IHC4	–	26	s.a	AChE4	–	19	p.r.d
EM5	46	9.5	m.a	IvMI 5	–	14	o.m.i		–			AChE5	–	24	p.r.d
EM6	60	10.5	m.a	IvMI 6	–	17	*Hydronephrosis*		–			AChE6	–	25	p.r.d
EM7	70	11.5	m.a	IvMI 7	–	22	*Carcinoma Cervicis uteri*		–			AChE7	–	26	p.r.d
EM8	83	12.5	m.a	IvMI 8	–	22	o.m.i		–			AChE8	–	26	s.a
EM9	100	13	m.a	IvMI 9	–	26	*Dilatatio ductus Biliaris*		–			AChE9	–	28	p.r.d
EM10	120	15	m.a	IvMI 10	–	29	*Cholecystolithiasis*		–				–		
EM11	210	22	s.a	IvMI 11	–	28	o.m.i		–				–		
EM12	216	23	s.a		–				–				–		
EM13	245	26	s.a		–				–				–		

-Postmortal period: within 4 h

-Source: specimens are part of Zagreb Neuroembryological Collection

-Cause of death: m.a.—medical abortion, s.a.—spontaneous abortion, p.r.d.—preterm respiratory distress, o.m.i.—other abdominal maternal indication

- +—specimens provided from Human Developmental Biology Resource

### Source 1

For electron microscopy analysis, tissue blocks (1–2 mm thick) oriented perpendicularly to the pia were dissected from coronal slabs containing the respective somatosensory area (postcentral gyrus in older or midlateral parietal area at the hippocampal level in younger specimens) of postmortem human fetal brains which were fixed by immersion and obtained after legal or spontaneous abortions (Table 1).

All specimens used in the electron microscopy analysis were collected within 4 h after death and were normal with regard to pathological findings and clinical histories. The tissue blocks (1 mm thick) were processed as described by Kostovic and Rakic (1990). EM analysis was performed in two steps on two sets of adjacent EM blocks. The ultrathin section from the first set of blocks was used to systematically analyze the morphology and classification of various presynaptic-postsynaptic morphologies of synaptic types, identified by the membrane-associated densities, synaptic vesicles and synaptic cleft (Table 2, first and second column) and non-synaptic cell junctions as characterized by membrane-associated densities only (Table 2, third column). Specimens listed in Table 2 were selected out of 27 fetal specimens based on quality of fixation. Ultrathin sections from all ages were inspected systematically by vertical probes (between 8 and 23 probes) at magnification 50,000x, and every synapse or junction with a known laminar position was EM micrographed and printed at a final magnification of 28,000x. Gaining a better overview of the surrounding neuropil in synapses containing cytoarchitectonic compartments required printing some sections at a magnification of 14,000x. The second set of EM-embedded blocks used for the quantification was adjacent to the blocks used for synapse classification and cut out from the same fetal specimens. 8 probes on two blocks for each specimen were performed on perfectly oriented ultrathin sections. These EM blocks were not previously used in our other publications (Kostovic and Rakic 1990).

**Table 2:** Number of synapses and non-synaptic junctions

Asymmetric synapses		Symmetric synapses		Non-synaptic junctions	
A1 = bouton-small dendrite	**172**	**S1**	**14**	**J1**	**84**
A2 = bouton-large dendrite	**21**	**S2**	**4**	**J2**	**3**
A3 = bouton-growth cone	**4**	**S3**	**0**	**J3**	**8**
A4 = bouton-proximal dendrite	**25**	**S4**	**8**	**J4**	**1**
A5 = bouton-soma	**10**	**S5**	**3**		
A6 = bouton-d. bifurcation	**2**	**S6**	**0**		
A7 = bouton-dendritic shafts	**14**	**S7**	**2**		
A8 = “en passante” a. varicosities-dendrite	**12**	**S8**	**0**		
A9 = double synaptic contact	**13**	**S9**	**0**		
A10 = axon-spine like	**5**	**S10**	**0**		
SUM	**278**		**31**		**96**
Total number of synapses and non-synaptic junctions					**405**

The designation of presynaptic and postsynaptic elements in the second column (symmetrical synapses, S) and designation of contacting elements (third column, J) corresponds to description given in the first column. For example S1 means symmetrical synapse between bouton-small dendrite

The synaptic locations are presented in the form of a histogram, which demonstrates the spatial distribution of synapses for a given developmental age. The histograms show the actual number of identified synapses found per 100 µm on the depth class (bins of equal width on the histogram).

### Source 2: histology and immunohistochemistry

Several human postmortem tissue specimens were used to obtain the data on the development of histogenesis of the prospective fetal somatosensory cortex based on existing material from the Zagreb Neuroembryological Collection collected since 1974 (AChE histochemistry along with adjacent Nissl-selected specimens and a short postmortem delay before fixation and non-neurological diagnoses). The list of cases is shown in Table 1. We also used four paraffin-embedded specimens from 10–26 PCW for immunohistochemistry. Only the brains of fetuses (10–26 PCW) without any sign of MR structural abnormalities and genetic abnormalities were included in the study. The developmental age of the specimen was expressed as PCW on the basis of crown-rump length (CRL) measurements. Some of the prenatal specimens were received from the Joint MRC-Wellcome Trust Human Developmental Biology Resource (HDBR), grant #099175/Z/12/Z, with declared ethical permission. A list of all analyzed cases is shown in Table 1.

AChE histochemistry was performed using Lewis’s modification of the Koelle–Friedenwald acetylthiocholine iodide method, as previously described (Kostovic and Goldman-Rakic 1983; Kostović 1986). In short, brain tissue was fixed for up to 24 h in 10% buffered PFA, frozen sectioned, followed by incubation in sodium sulfide in 0.2 M acetic acid until the reaction product was developed (up to 24 h), and cover-slipped. The controls to show the specificity of AChE were performed using inhibitors, as described (Kostovic and Goldman-Rakic 1983). Images were taken using a digital histological slide scanner, NanoZoomer 2.0RS (Hamamatsu, Japan).

For the purpose of performing immunohistochemistry, the postmortem brain tissue was fixed in 4% paraformaldehyde (PFA) and 0.1 M phosphate-buffered saline (pH = 7.4), tissue blocks were paraffin-embedded and sections (10 μm) cut on a microtome. Immunohistochemical staining was done using our standard laboratory protocol (Žunić Išasegi et al. 2018; Kopić et al. 2023) utilizing the following primary antibodies: anti-SNAP-25 (Biolegend, 836,301, 1:1,000); anti-Synaptophysin (DAKO, m-7315, 1:100); and Fibronectin (Sigma, F3648, 1:200) (Table 3). Positive immunostaining was visualized using SIGMAFAST™ DAB with Metal Enhancer. Sections were mounted with Poly-Mount (Polysciences, Inc.) and cover-slipped.

**Table 3:** List of antibodies

Antibody	Supplier and catalogue number	Host	Antibody Concentration
Anti-SNAP25	BioLegend, 836,304	Mouse monoclonal	1:1000
Anti-SYN	Dako, M7315	Mouse monoclonal	1:50
Anti-FN	Sigma, F3648	Rabbit polyclonal	1:200

Finally, images were taken using a digital histological slide scanner NanoZoomer 2.0RS (Hamamatsu, Japan) and assembled in the Microsoft Publisher.

### Source 3: in vivo MR

For the in vivo part of the study, 11 in vivo and in utero fetal MR scans (age range 11–29 PCW) were acquired during the medically indicated diagnostic examination of the pelvic region in pregnant women (maternal indication listed in Table. 1, second column showing MR specimens) and only fetuses with no signs of any pathology were included in the present study. Fast T2 weighted half-Fourier single-shot turbo spin-echo (HASTE sequence) MR imaging was performed on a 1.5 Tesla device (Magnetom Symphony Siemens at the University Hospital Centre Zagreb, Zagreb) by experienced neuroradiologists (Prof. Marko Radoš). All in vivo MR images are now part of the Zagreb Developmental MR Collection, which also includes a cohort of more than 200 prematurely born infants who underwent longitudinal follow-up MR examinations and a large number of postmortem MR images of the human fetal brain (Kostović et al. 2014). The postmortem scans from our MR repository served as an ad hoc reference for confirming laminar patterns described in the present study.

The final alignment of histograms showing the spatial distribution of synapses, adjacent 1-micron Nissl stained sections, immunoreacted histological sections and in vivo in utero MR images was performed at the same micrometer scale using the pial surface as an approximate superficial landmark, while the superficial and deep border of the cell-dense cortical plate and external border of the intermediate zone served for achieving alignment with poorly defined landmarks on the MR. Given that delineation of the cortical plate in the youngest specimen (10.5 PCW) was not possible, we used several age-matched MR in vitro postmortem specimens to confirm that a cell-dense cortical plate can serve as a reliable laminar landmark in the fetal pallium (Radoš et al. 2006).

The borders of the cortical plate were also helpful for juxtaposing 1-micron semi-thin (EM) sections (almost no shrinkage) and paraffin-embedded blocks (notable shrinkage), providing a surprisingly accurate comparison between different immunoreacted histological sections, MR images and delineation of synapse-rich compartments.

The resolution size of 1.5 Tesla MR does not permit delineation of the marginal zone.

## Results

The major aspects of the results are described in two separate paragraphs: Section I. Classification of synaptic junctions based on the morphology of presynaptic and postsynaptic elements, and Section II. The spatial distribution of synapses within the cortical compartments at different developmental phases correlates with the distribution of immunocytochemical markers and the lamination pattern on in vivo MR images. Identification and classification of different types of synaptic junctions (Section I of the Results) was the first step in our study. All types of synaptic junctions classified as synapses displaying membrane-associated densities, synaptic cleft, and vesicles inspected during this first step of analysis were later counted in different sets of EM blocks in vertical probes, and their laminar distribution was presented as a function of subpial depth (in histograms presented in Section II of the Results).

The developmental periods presented correspond essentially to phases defined by cytoachitectonic, chemoarchitectonic and cell dynamics criteria defined in our previous studies (Kostovic and Rakic 1990; Kostović et al. 2019a): (1) Primary condensation of the cortical plate or pre-subplate phase 8–12 PCW; (2) Formation of the second cortical plate or the subplate formation (expansion) phase 12–14 PCW, (3) Secondary condensation of the cortical plate or phase of the increase in the subplate thickness 15–18 PVW; and (4) Stationary (maximal) subplate phase with the first lamination within the cortical plate 22–26 PCW. In the youngest specimen examined using EM (CF 71, 7.5 PCW) before the formation of the cortical plate (preplate phase), we did not find synapses, as described by Larroche 1981; this specimen was not included in the study.

**Section I.** Classification of synaptic junctions based on the morphology of presynaptic and postsynaptic elements.

To classify the different types of synaptic junctions, we have performed a systematic analysis of printed electron micrographs, at a final magnification of 28,000x, obtained during systematic vertical screening across the entire thickness of the fetal cortex on ultrathin sections from EM blocks cut perpendicularly to the pia, and obtained from fetal specimens at different age groups with a known subpial depth as determined by its juxtaposition to the adjacent 1-micron thick Nissl stained plastic sections.

All junctions identified as synapses in the present study display membrane-associated densities, visible cleft and vesicles in the pre-synaptic element. This identification criterion corresponds to the criterion of DeFelipe et al. 1999 as well as grade 1 and grade 4 posed by Molliver and Van der Loos 1970 and Molliver et al. 1973. We classified and counted junctions with only more than two vesicles and a visible synaptic cleft. Thus, the category of unclassified synapses (for example, grade 2 and grade 3 given by Molliver and Van der Loos 1970 was not counted in the present study. Based on the identification of presynaptic and postsynaptic elements, the synapses were classified as asymmetrical and marked as A1-A10 and symmetrically marked with S. Table 2. lists all synaptic types found in the present study. In contrast to the 10 types of asymmetrical synapses (Table 2, first column) the symmetrical synapses form only six types of junctions between the presynaptic and postsynaptic elements (S1–S5 and S7, Table 2 second column).

**Fig. 1:**
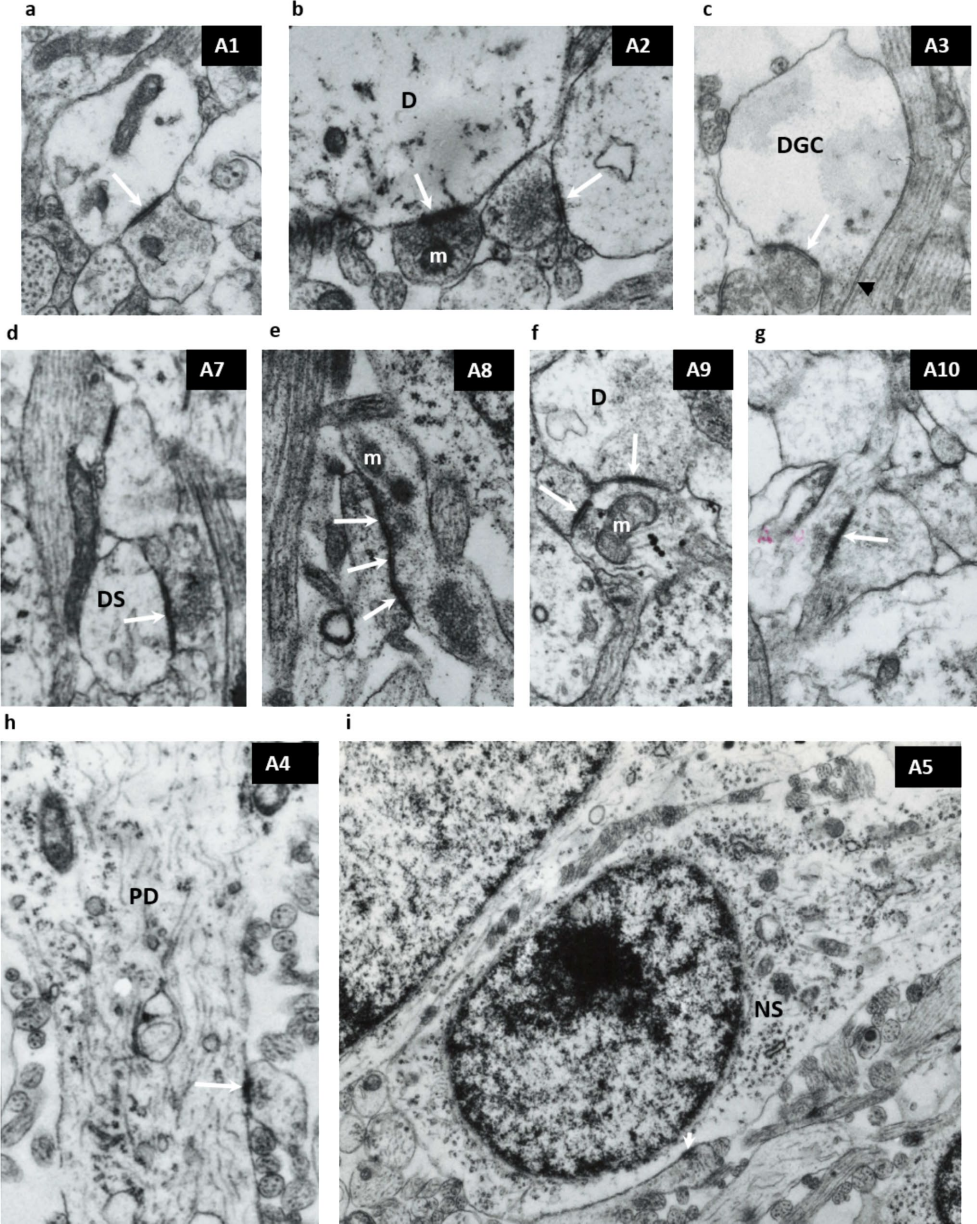
Asymmetrical synapses in fetal SP, MZ and CP. White arrows indicate postsynaptic element. Capital letters with numbers (**A1–A10**) designate asymmetric synapses listed in Table 2, which shows their frequency of appearance within the total sum of asymmetric synapses. **a** Shows most frequently observed synapse between terminal axonal bouton and small dendrite in the deep subplate of 24 old PCW old fetus. **b** Is contact between bouton and large dendrite (SP, 24 PCW). **c** Shows synapse on dendritic growth cone (DGC). Arrowhead indicates thin neck of growth cone. SP-CP interface, 24 PCW **d** is a synapse in the CP in specimen 26 PCW old fetus showing contact of axons with dendritic shafts. **e** Shows varicosities of axon terminal forming “en passant” synapse (SP-CP interface 24 PCW), **f** axonal bouton making double synaptic contacts with 2 dendritic profiles (CP 24 PCW), **g** synapse in deep portion of CP specimen showing contact of axon on spine-like projection of the prospective dendrite (24 PCW), **h** synapse between bouton and proximal dendrite (PD) of SP neuron in 24 PCW old specimen.** i** Asymmetric synapse between bouton and soma (NS) of the SP neuron in the transitional zone between SP and CP (24 PCW) specimen. Note that many presynaptic terminals contain mitochondria (**m** on **b, e, f**). Synaptic vesicles are clustered in the proximity of presynaptic membrane associated densities. Magnification × 28 000 for (**a–g**), × 14 000 **h, i**

**Fig. 2:**
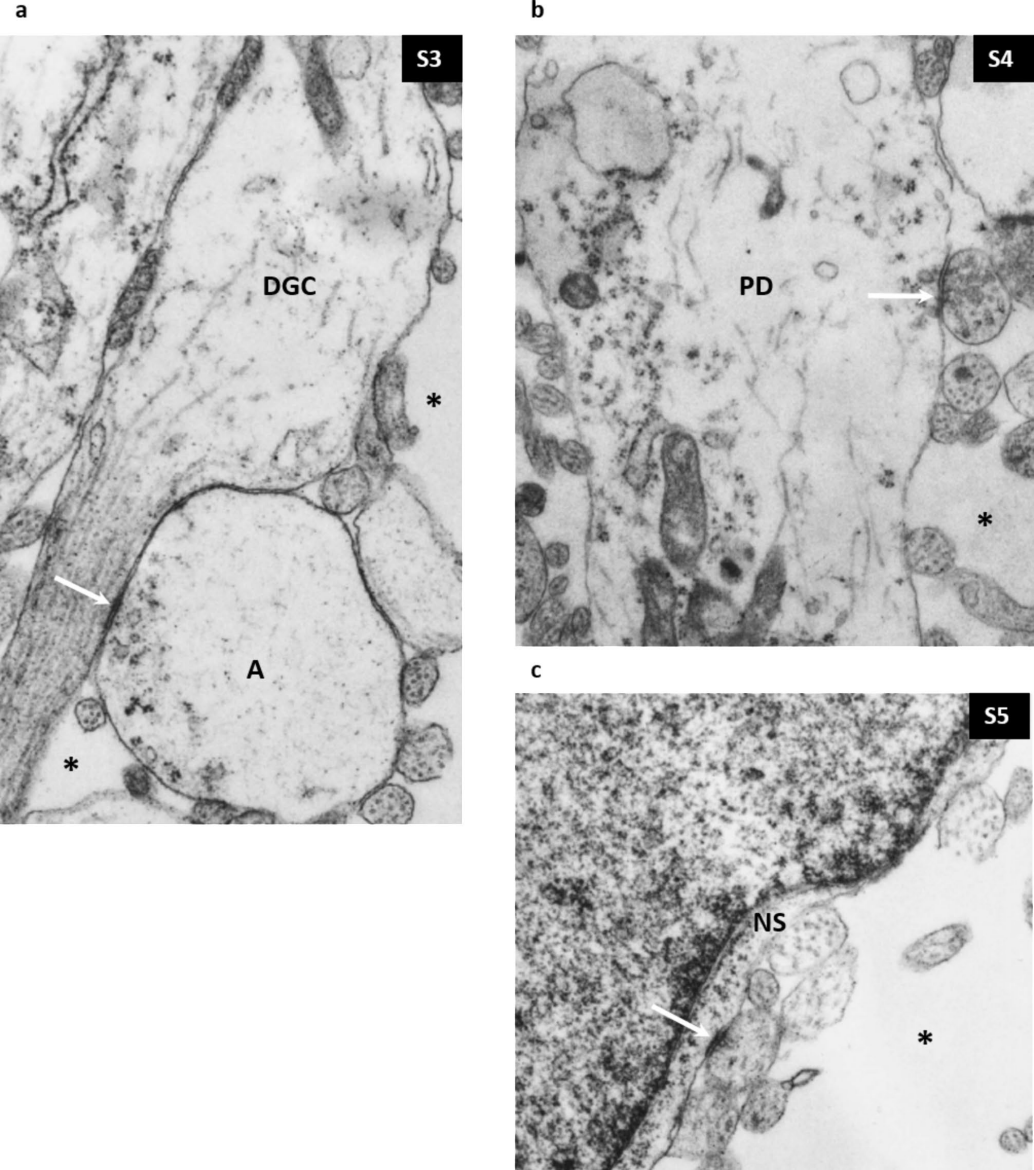
**a** Shows symmetrical synapse between large swollen axonal terminal (**a**) and dendritic growth cone (DGC) within SP compartment of 24 PCW old specimen. **b** Typical symmetric synapse on proximal dendrite (PD) of SP neuron in 24 PCW old specimen. **c** axosomatic symmetrical synapse on the perikarion (NS) of SP neuron in a 15 PCW old specimen. Abundant ECM (asterisk on (**a**–**c**) surrounds synaptic elements. Magnification × 28,000 for (**a)**, × 14,000 for (**b, c**)

The asymmetrical synapses (labeled as A in Table 2) are shown in Fig. 1a–i, while Fig. 2a, c shows three types of the most frequently found symmetrical synapses (labeled as S in Table 2). The asymmetrical axodendritic synapse classified as type A1 shows the contact between the axon terminal (bouton) and small dendrite (Fig. 1a). The presynaptic axon terminals and dendrites frequently show mitochondria and other organelles. A similar morphology is present in the A2 type of synapses where the postsynaptic dendrite has a larger diameter (D in Fig. 1b) and has a “swollen” appearance. The A3 type of synapse is in the contact between the axon terminal and dendritic growth cone (DGC in Fig. 1c). The dendritic growth cone is recognizable by its thin “neck” (arrowhead on Fig. 1c and enlarged pale and “swollen” cytoplasmatic content with very few organelles (DGC in Fig. 1c). Type A7 is found in the cortical plate where postsynaptic elements are elongated and predominantly vertically oriented dendritic “shafts” (Fig. 1d, see also Fig. 12b where radial dendritic shafts are marked as DS). Asymmetrical synapses between varicosities of preterminal axons and dendrites of deep cortical cells (A8 type) resemble “en passant” synapses, as shown in Fig. 1e. In older ages, the bouton-like type of axonal terminals occasionally makes double contacts (type A9) and are mostly found in the compartments with higher synaptic density, such as MZ or superficial SP (arrows on Fig. 1f). In the oldest examined specimen, some synaptic junctions in the cortical plate are found on the occasional spine-like protrusions from shafts of the pyramidal neurons (type A10 in Fig. 1g). Asymmetrical synapses on proximal dendrites of SP neurons (PD in Fig. 1h) are classified as type A4. Although most axosomatic synapses are symmetrical, occasionally, small axonal boutons can create asymmetrical synapses on cell perikarya of deep cortical neurons (type A5 in Fig. 1i).

Symmetrical junctions are significantly less numerous and show less variability. Figure 2a shows an interesting synapse on the growth cone. Figures 2b, c show frequently observed contacts among symmetrical synapses: an axosomatic symmetrical synapse on the subplate neuron (NS in Fig. 2c showing a S5-type synapse) and synaptic junction between axon terminal (bouton) and the proximal dendrite of the subplate neuron (PD in Fig. 2b).

**Fig. 3:**
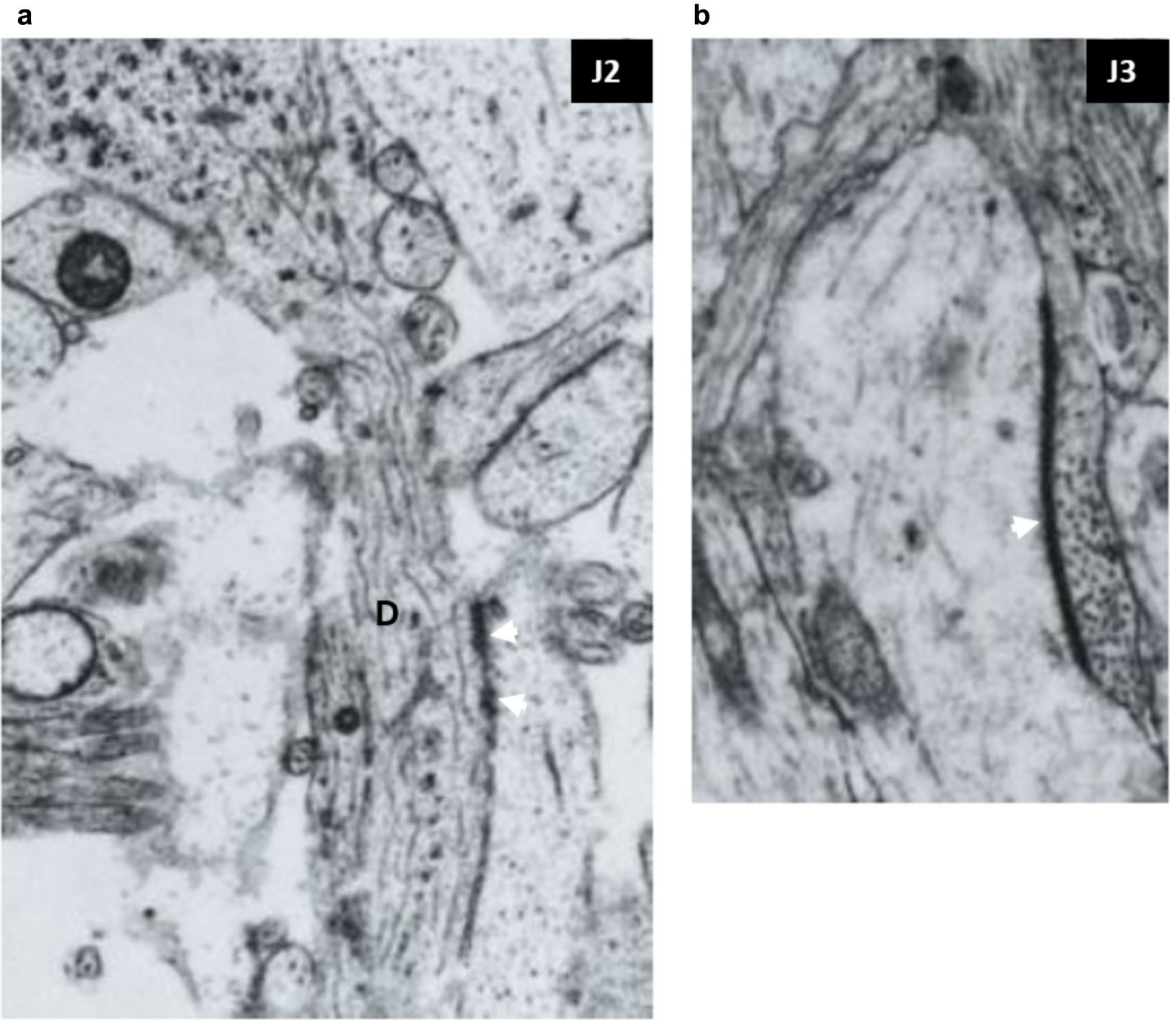
Non-synaptic interneuronal contacts (cell junctions) with membrane-associated densities. **a** Shows puncta adherentia (arrowheads) between axon and dendrite (D) (presubplate 9.5 PCW). **b** Typical long intercellular junction (arrowhead) with thick membrane associated densities. (Marginal zone 24 PCW). Magnification 28,000×

Non-synaptic junctions. Numerous non-synaptic junctions were found in all layers and across all ages studied. The most frequently found non-synaptic junctions were simple contacts with membrane-associated densities between small profile processes (puncta adherentia) (Peters et al. 1970). Figure 3a shows multiple puncta adherentia (type J2) connecting axon and dendrites (D in Fig. 3a) while Fig. 3b shows characteristic long contact between axon and dendrite shown in Fig. 3b, which is classified as a J3-type junction (Table 2). Intercellular junctions were also found between endfeet of glia in the marginal zone characterized by multiple membrane-associated densities (type J2, Table 2, shown later in Fig. 8).

**Fig. 4:**
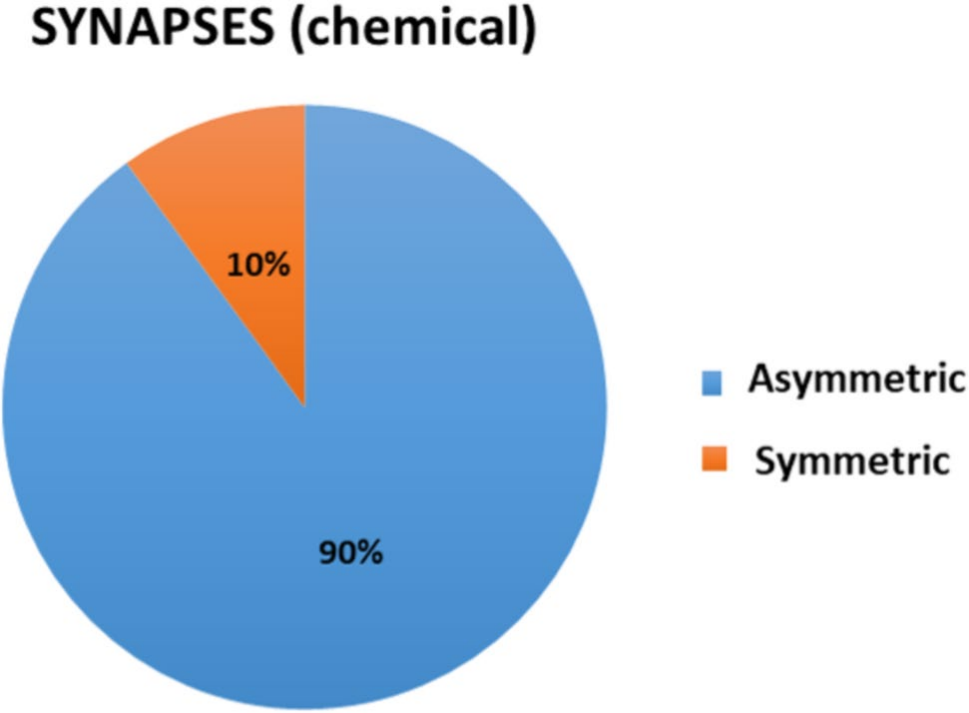
Percentage of asymmetric (blue) and symmetric (orange) synapses found in total population of 309 recorded synapses. Predominance of asymmetric synaptic junction is obvious

**Fig. 5:**
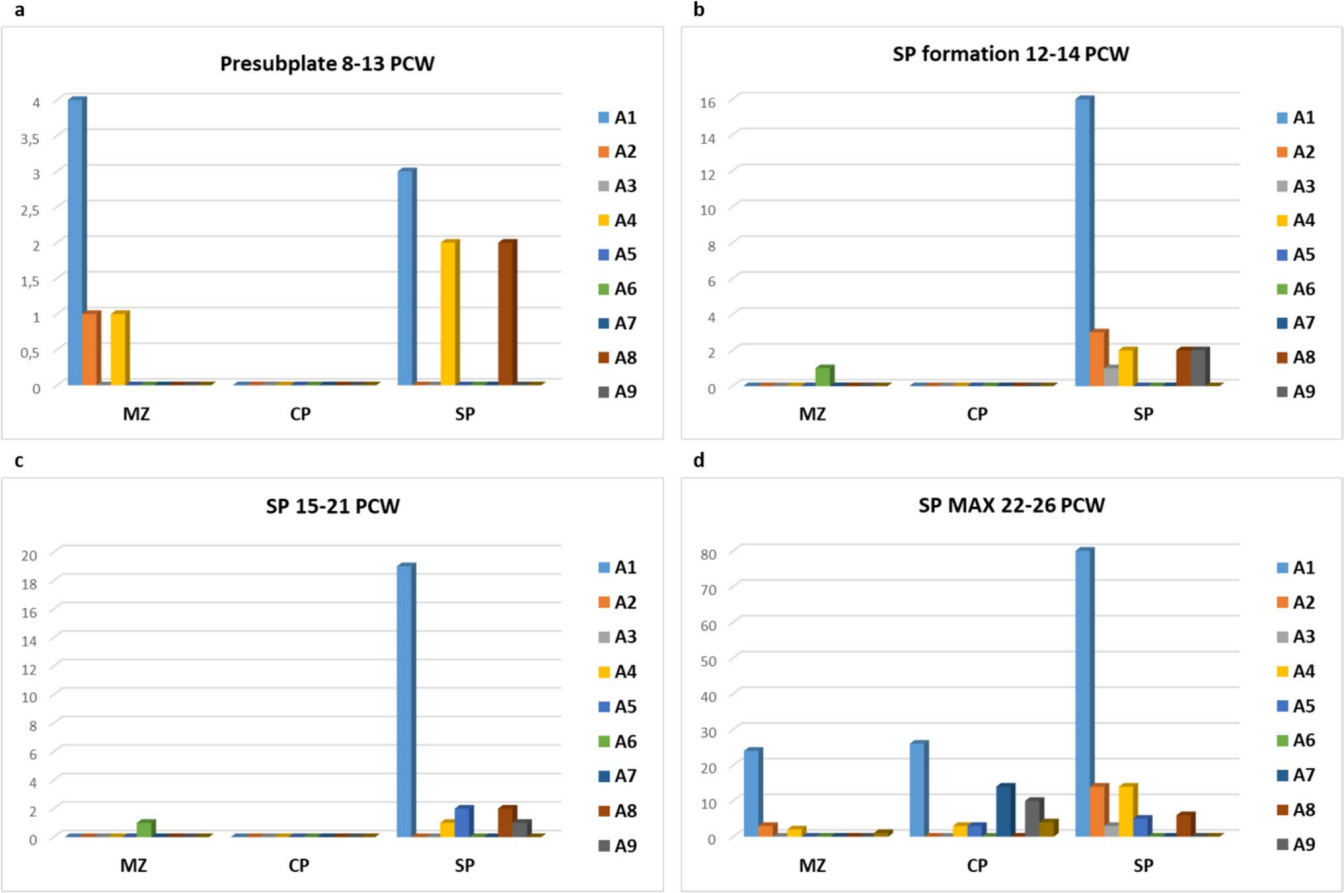
Histograms showing relative proportions in number of different types of asymmetric synapses in presubplate (**a**), subplate formation (**b**), SP (**c**) and subplate maximum phase (**d**). In all phases examined the predominant type of synaptic contact is between the terminal bouton and small dendrites (A1 type-light blue). Note that CP is free of synapses until subplate maximum phase. During subplate maximum phase synapses appear also on the dendritic shafts of the pyramidal neurons in the CP (A7 dark blue). Presence of synapses on proximal dendrites (A4) and somata (A5) of the subplate neurons proves postsynaptic nature of the subplate neurons. Note also that few synapses were found on the bifurcation of the apical dendrites (A6 green) which is specific characteristic of the MZ compartment

**Fig. 6:**
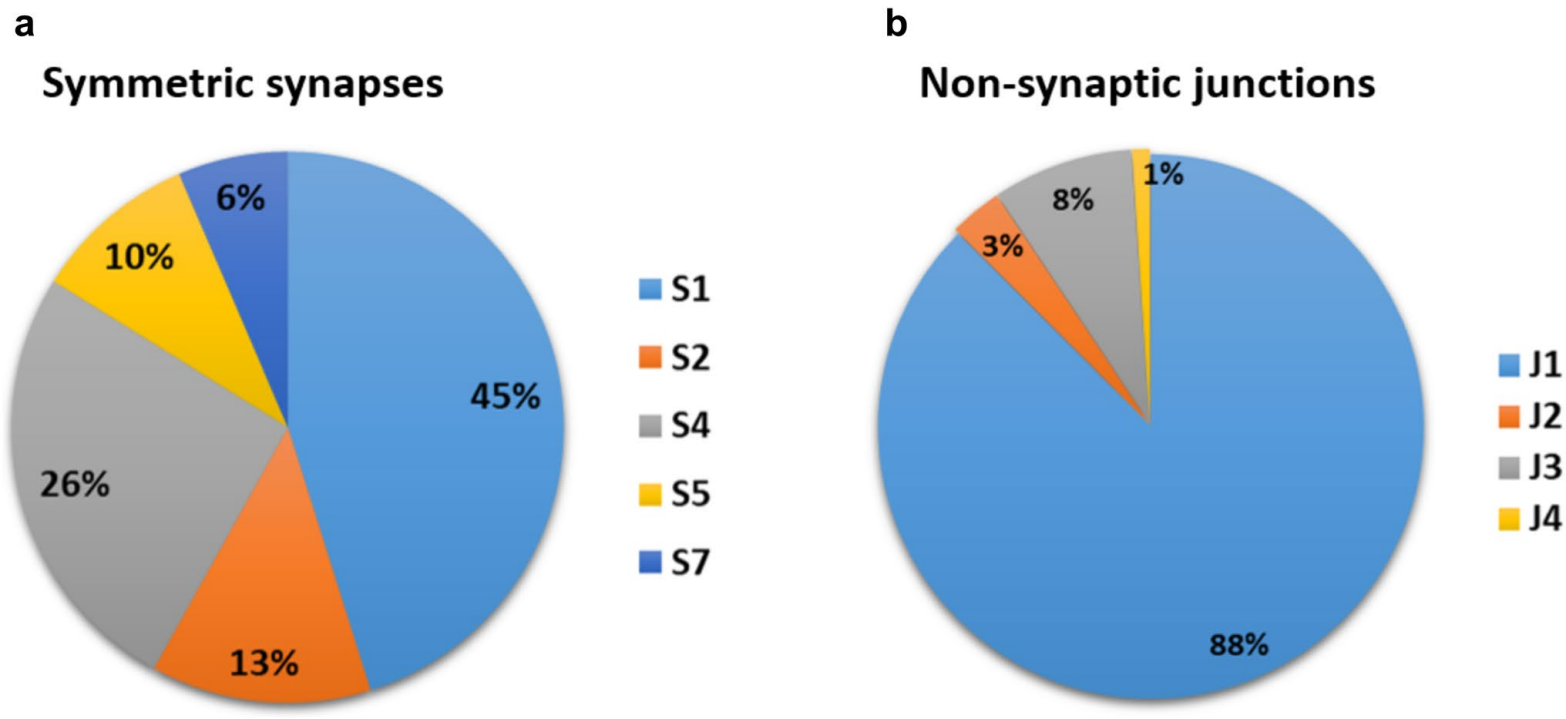
Graphical representation of different symmetric synapses types of percentage in total population recorded. Note that the two types of symmetric synapses prevail: synapse on small dendrite (S1 light blue) and synapses on proximal dendrite (S4 dark grey). Note relative high percentage of synapses on large dendrite (S2 orange) and relatively small percentage found on soma (S5 yellow). Synapses on dendritic shafts are rather rare (S7 dark blue). **b** Non-synaptic cells are readily found in all compartments but there are no typical gap junctions. The most numerous (J1-blue) are puncta adherentia. Long membrane density appositions are found in 8 percentage of junctions (J3 dark grey), while multiple junctions (J3) are shown in orange. Non-synaptic junctions on the proximal dendrite are very rare (J4 yellow)

After the classification of the observed synapses (shown in Figs. 1, 2), we estimated the following: (1) The percentage of asymmetrical and symmetrical synapses in the total population (309) of the recorded synapses (Fig. 4); (2) The number of different asymmetric synaptic types (A1–A9) found in each transient compartment and each developmental age in the analyzed histograms (Fig. 5a–d) using subpial depth and cytoarchitectonic landmarks as parameters for compartmental inclusion and morphological type (for type classification see Table 2) of the whole population of synapses recorded. 3. Percentage of the different types of symmetric synapses and non-synaptic junctions recorded (Fig. 6a, b). The main result of this classification is that 91 percentage of all synapses are asymmetric if one takes into account all phases of the development and all examined compartments (Fig. 4). Given that the vast majority of synapses are asymmetric, the relative frequency at different phases and different compartments were presented on histograms only for this type of synapses (Fig. 5a–d). These histograms show the relative number of characteristic types of synapses in the subplate (SP) and the marginal zone (MZ) for all four phases of the development (Fig. 5a–d). The predominance of type A1 (small presynaptic axon terminal-postsynaptic small dendrite) in the subplate compartment is evident. The observed differences between compartments and ages shown on histograms are referred to and incorporated in the description of the synaptic distribution of each developmental phase (Fig. 5g, presubplate; Fig. 5b, subplate formation; Fig. 5c, midfetal subplate; Fig. 5d, subplate maximum).

It is important to note that synaptic types of the cortical plate appeared only in older specimens (22–26 PCW) since no synapses were found in the CP of the 15 PCW old fetus (specimens between 16 and 21 PCW were not fixed for EM analysis, Table 1).

After 22 PCW, synapses appear frequently on the dendritic shafts and small dendrites (Fig. 5d, blue bars). Typically, but rarely observed are synapses situated on the main apical dendrite bifurcation when entering the marginal zone (Fig. 5, green bar).

The percentage of symmetrical synapses is shown in Fig. 6a. Of symmetric junctions, 45% are located on small dendrites (Fig. 6a, blue), while 26% are located on the proximal dendrite (Fig. 6a, dark grey). A relatively small percentage of symmetrical synapses is found on cell somata (Fig. 6, yellow). The percentage of synapses on large dendrites (S2 orange) is relatively high.

The percentage of different types of non-synaptic intercellular junctions is shown in Fig. 6b. The majority (88% of non-synaptic intercellular contacts) is a “simple” type, which corresponds to puncta adherentia (Peters et al. 1970). The intercellular long, dense membrane apposition contacts are relatively frequently observed (J3 in Fig. 6b). Typical gap junctions were not found in specimens examined in this study.

**Section II.** The spatial distribution of the synapses within cortical compartments at different developmental phases correlated with the distribution of the immunocytochemical markers and pattern of in vivo MR lamination.

### Presubplate phase—primary condensation of the cortical plate

**Fig. 7:**
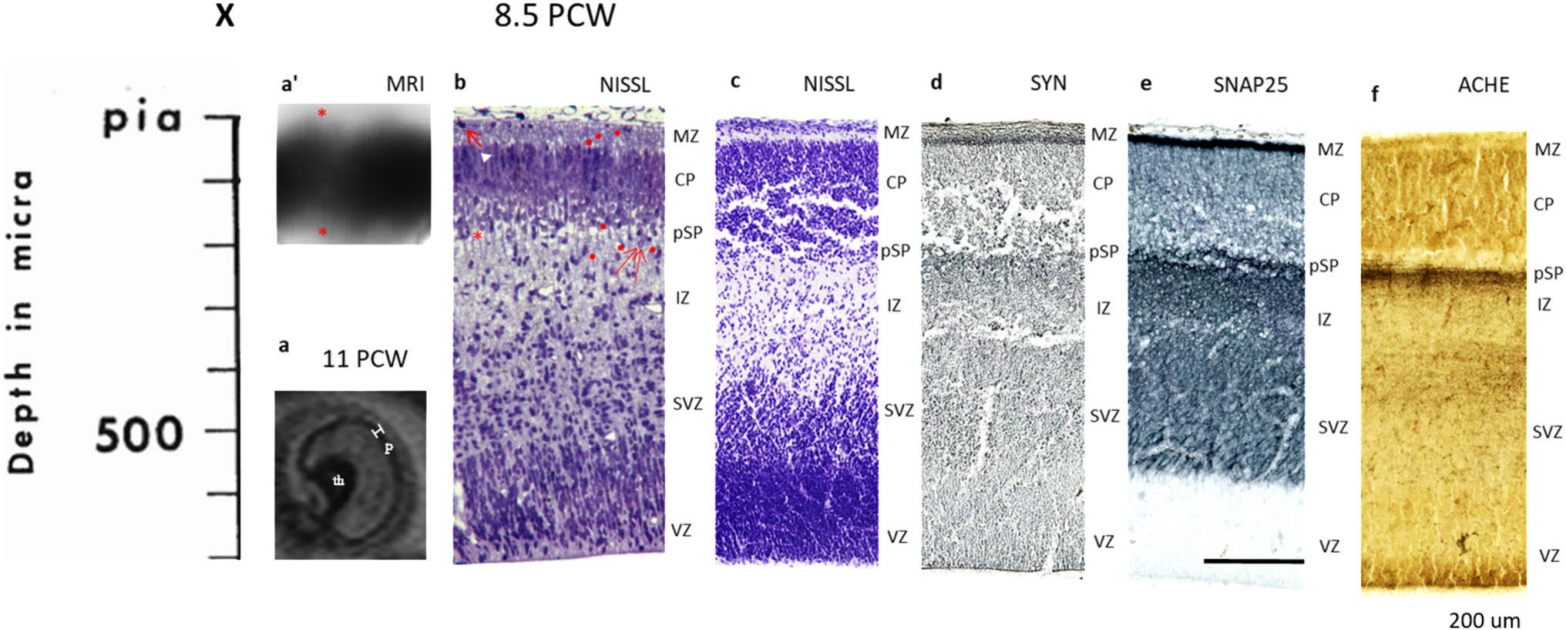
Laminar distribution of synapses during pre-subplate phase (8–11 PCW). Few synapses above and below cortical plate (**B**) red puncta shown on adjacent 1 micron Nissl stained plastic section). **a** In vivo MR: lamination of pallium is poorly visible (p = pallium, th = thalamus). Vertical stripe of the cerebral wall (bounded bar is shown on high power magnification on (**a**). Thickness of the cell dense T2 low signal band of pallium corresponds approximately to the thickness of the CP shown on Nissl stained plastic section of two weeks younger specimen (**b**) but from same developmental phase (pSP). Nissl stained plastic section shows few large cells in pSP (double arrow), prospective Cajal-Retzius cells in MZ (single arrow), embryonic collumns (white triangle) and enlarged ECM (asterisk). On figure **a'** low T2 signal intensity band is delineated on both sides with higher signaling intensity laminae (asterices). Presubplate is also visible on histological sections stained with Nissl (**c**) or immunoreactive with synaptic and fibrillar markers as disrupted narrow zone (pSP). Synaptophysin reactive (**d**), SNAP25 reactive (**e**) and above AchE reactive fibres (**f**) are situated below pSP. Sublamination of MZ is visible on Nissl (**b**, **c**) and immunoreactive sections (**d**, **e**). **c**–**e** Show immunoreacted sections from a single brain. Scale showing depth of EM inspected tissue corresponds to 1 µm plastic Nissl from (**b**) is marked with X above the scale. Area of inspected tissue is 33,600 µm^2^ Scale bar for IHC stainined tissue is 200 microns on (**e**)

The fetal cerebral wall in the prospective somatosensory area is composed of the following cytoarchitectonic compartments (from pia to the ventricles): marginal zone (MZ), fibrillar lamina containing large Cajal-Retzius cells (Fig. 7b, arrow), cortical plate (CP)–densely cell packed lamina composed of vertically arranged embryonic columns (Fig. 7b white triangle), PSP (presubplate)–disrupted, extracellular matrix (ECM) containing a layer (Fig. 7b, asterisk) with few large cells (Fig. 7b, double arrow), intermediate zone (IZ)–composed of the bundles of axons and migratory neurons (Fig. 7b), subventricular zone (SVZ)–composed of loosely arranged polymorphic cells, ventricular zone (VZ)–composed of radially arranged cells with the mitotic figures close to the ventricular surface. On the basis of the neuronal morphology (postmigratory type of dendritic branching) or ultrastructural features (presence of synapses), we consider that three superficial compartments (MZ, CP, PSP) form the anlage of the fetal cortex (Kostovic and Rakic 1990; Meyer et al. 2000).

Synapses are present in small numbers in two compartments: MZ (above the CP) and in PSP (below the CP). The location of synapses found in all vertical probes (from the pia to the ventricle) were marked (Fig. 7b, red dots) on a 1-µm Nissl-stained plastic section adjacent to the ultrathin section examined at the EM level. Based on the fact that the distribution of these earliest synapses was restricted to two compartments (above and below CP), this phase of synaptogenesis is described as an onset of the two-compartmental phase.

**Fig. 8:**
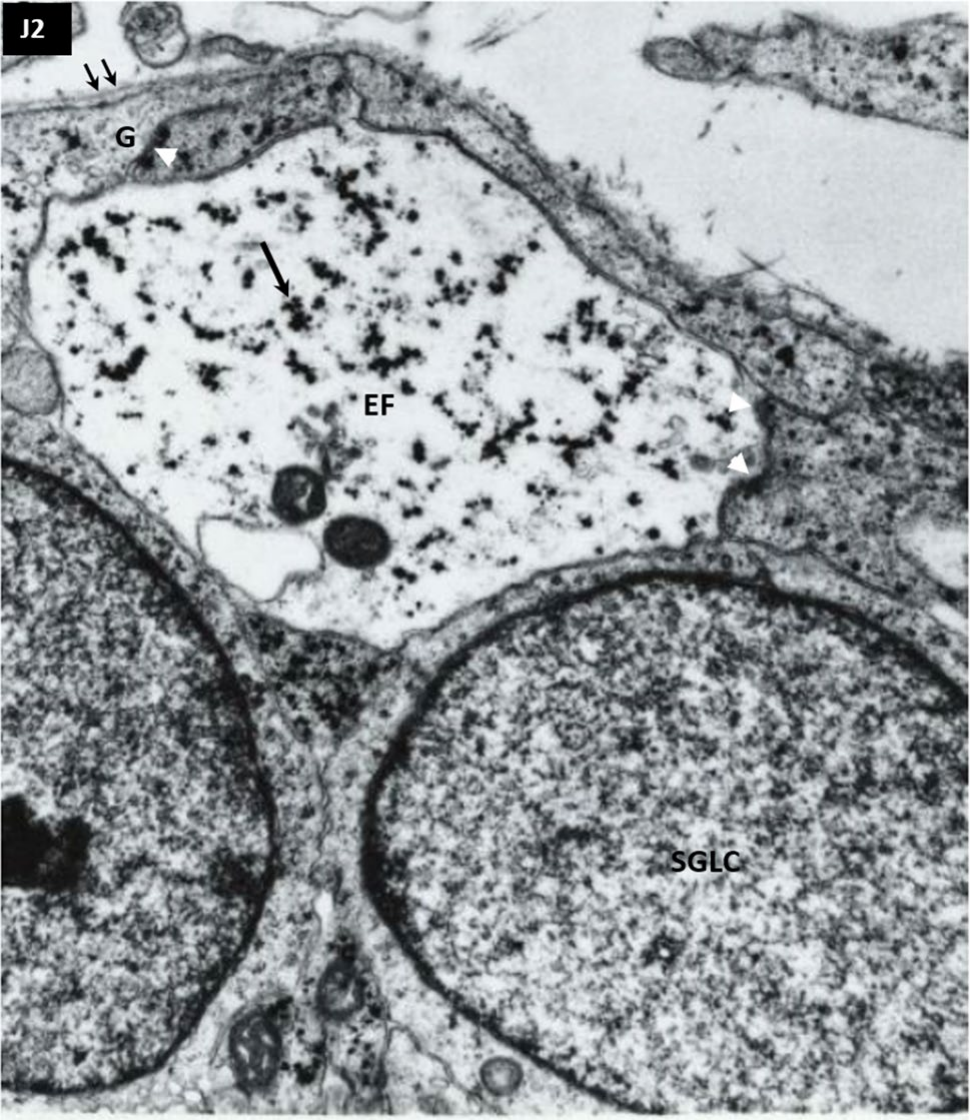
Glial endfeet (EF) (Light structure with glycogen granula-arrow, in the middle) and other cellular profiles on early established mesenchymal-neuroepitelial interface. Note puncta adherentia and long intercellular contacts between glial cells (arrowheads). Basal membrane is indicated with double arrow. First alignment of small MZ cells into subpial granular layer (SGLC), 11.5 PCW, magnification 28,000×

The majority of synapses found in the marginal zone were asymmetric, located on the postsynaptic elements resembling ultrastructural features of small dendrites (type A1 of the classification, Fig. 1A). No synapses were found in the cell-dense cortical plate. Synapses in PSP were mostly axodendritic (type AS1), but notable are several asymmetric and symmetric synapses found on the proximal dendrites of PSP neurons (type A4 in Fig. 1 and S4 in Fig. 2). Neuropil of the MZ is fibrillar with numerous dendrites, disrupted ECM areas and densely packed endfeet contacting the basal membrane. Prominent low-density (light) endfeet (EF on Fig. 8, is loaded with glycogen granules (Fig. 8, arrow). The first subpial granular layer (SGL) cells start to aggregate in the superficial portion of the MZ, showing immature perikarya with a poorly developed reticulum, which is in contrast to the elaborated, “machinery-like” cytoplasmatic reticulum of Cajal-Retzius cells. Numerous processes contact the basal membrane (Fig. 8, double arrow) and presumably belong to the glial cells and display intercellular junctions (Fig. 8, white arrowheads).

Immunocytochemically treated sections show that strongly synaptophysin and SNAP-25 reactive fibers, which form the tangential band (Fig. 7d, e), correspond to the subpial depth location of synaptic dots (Fig. 7b, red dots) in the marginal zone. The synapse-free CP does not show immunoreactivity for synaptic markers. Below the CP is another thin synaptic compartment within the PSP where transitioning with the superficial IZ (Fig. 7b, asterisk). Some synaptophysin and SNAP-25 immunoreactivity correspond to this synaptic lamina. However, the much stronger reactivity of synaptic markers is in the fibrillar intermediate zone (IZ in Fig. 7d, c), which also contains AChE reactive fibers originating in the thalamus (Kostovic and Goldman-Rakic 1983) and basal forebrain (Kostović 1986) as shown on Fig. 7f. In addition to synaptic markers presented on our figures we have compared our light microscopical delineation of SP with laminar pattern of distribution of SP markers presented by Wang et al. 2010 and Molnár and Clowry 2012. mRNA translation of synaptic marker CELF4 served us as an additional criterion for synaptic strata (Salamon et al. 2023).

Exact in vivo MR correlates of deep, synapse-rich compartments cannot be determined in this early phase due to the T2 low signal intensity throughout the cerebral pallium (p in Fig. 7a) appearing as a single, cell-dense band and pSP being too thin to be visualized. This situation is in contrast with in vitro MR imaging with long exposure time, which shows a laminated organization of the cerebral wall already in these early ages (Radoš et al. 2006). The in vivo MR image shown at “higher” (enlarged) magnification (Fig. 7a’) confirms that a cell-dense T2 low signal band forms in the pallium, which in the “subpial” location and thickness corresponds to the Nissl-stained 1-micron thick plastic section of a somewhat younger specimen from the same cortical region. Below the cell-dense CP band is a zone of high T2 signal intensity (asterisk), which probably corresponds to an ECM-rich intermediate compartment and above the low-signal pallial band is a similar T2 intense (“watery”) signal, which is space rich in the pericerebral fluid (Fig. 7a’ superficial asterisk). This change in signal intensity allows delineation between pallium and mesenchymal coverage of the fetal head.

### Formation of the second cortical plate or subplate formation (expansion) phase at 12–14 PCW

**Fig. 9:**
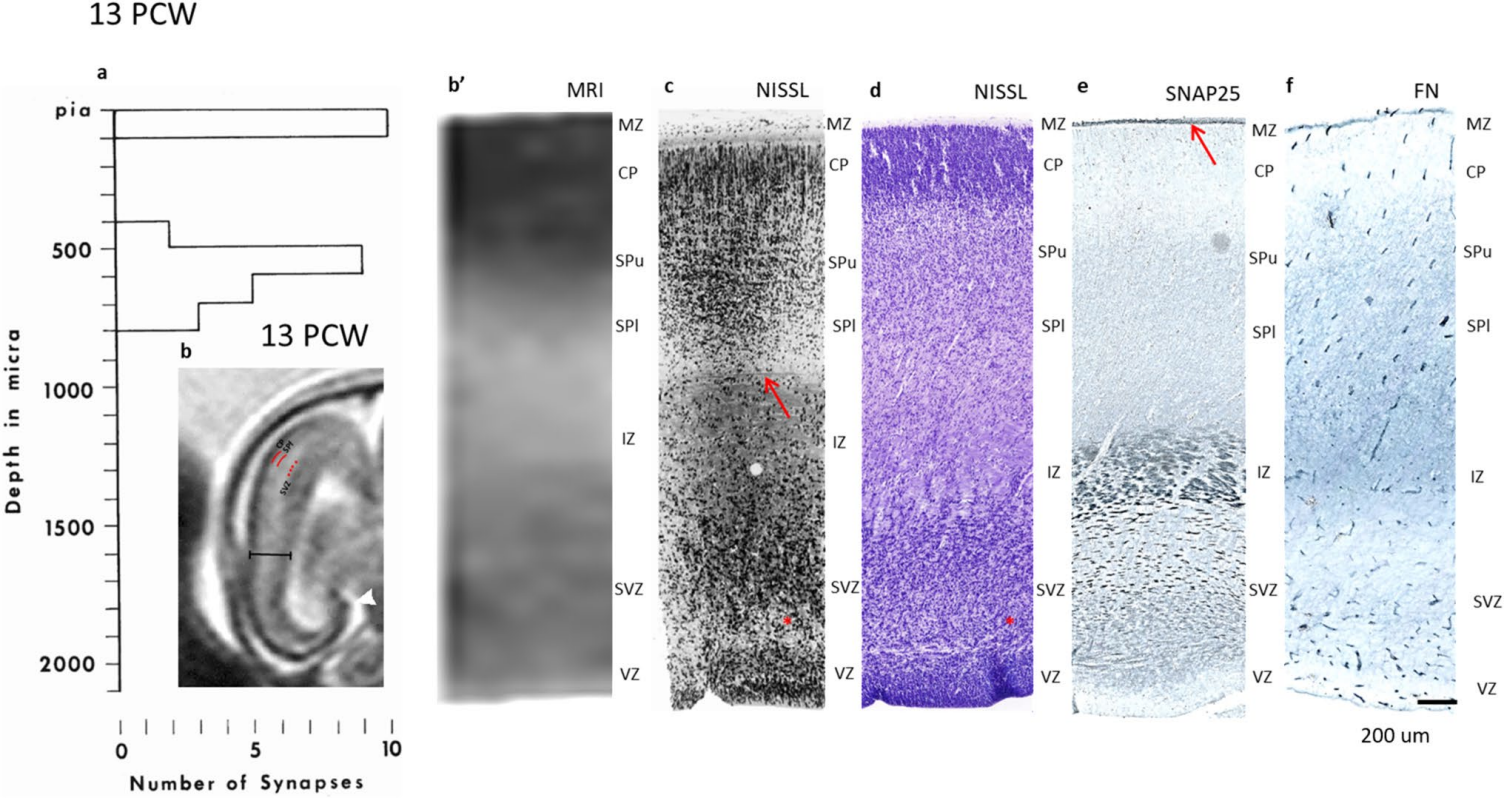
During phase of SP formation (“second plate”) (12–14 PCW) bi-compartmental distribution of synapses (**a**) shows spatial correlates with in vivo MR (**b** and **b’**) and light microscopic laminar pattern (**c–f**). Synapses are present in second plate or subplate upper (SPu) and this compartment is indicated on MR in vivo image between red lines. Histogram (**a**) on Fig. shows that synapses of two compartmental pattern are also found in deep, cell loose CP (SPu, SP formation phase), pSP—SPI. On MR in vivo image (low power MR is inserted into the histogram above apscisse) border between SVZ and IZ is marked with red dotted line. Delineation of cytoarchitectonic landmarks is less obvious on high magnification (**b’**) when MR scale is mached to magnification of 1 µm plastic Nissl stained section (**c**) but gradual transition of signal intensity from low signal CP towards higher signal SP. Lower SP (SPl on (**b’**)) and deeper situated IZ is discernible even at this level of scale magnification (**b’**). The whole thickness of cerebral wall is marked with black bar on low magnification of in vivo MR (**b**). CP, SP in formation and SVZ are marked with black capital letters. Border between IZ and SP is visible on Nissl stained 1 micron plastic section (**c**) indicated by red arrow; asterisk shows fibre-rich border between VZ and SVZ (**c**, **d**). It is interesting that synaptic marker SNAP25 shows fibres in IZ (**e**) while synapse containing SPu, and SPl zone are less reactive. This is in contrast with strongly reactive fibre rich zone in marginal compartment (**e**, arrow). Extracellular matrix immunoreactivity on fibronectin show absence of staining in CP and moderate activity in SPu, SPl and IZ. **c**–**f** Show immunoreacted sections from a single brain.Magnification scale in micra on histogram corresponds to magnification on plastic section **c** and approximately to magnification of (**d**–**f**). Area of inspected tissue is 100,800 µm^2^. Scale bar for IHC stainined tissue is 200 microns (**f**)

This phase of cortical development is very distinct in the human fetus at this age. The deep portion of the CP becomes gradually loose and merges with the cell-poor PSP layer, forming a new “expanded” cytoarchitectonic compartment–the SP (Fig. 9c, d). The loose “second” plate is called the upper subplate (SPu), whereas the deeper part of the subplate that merges with the former pSP forms the lower SPl (Fig. 9c, d). This terminology is consistent with the description in the humans and non-human primates (Kostovic and Rakic 1990; Duque et al. 2016). The superficial part of the CP is cell dense, the MZ shows sublaminar organization, and the whole thickness of the prospective somatosensory cortex can be delineated from the underlying IZ (Fig. 9c, arrow), which shows tangentially oriented bundles of axons (Fig. 9c, deeper to the red arrow).

During this phase, there is an increase in the number of synapses above and below the cell-dense part of the CP, and the quantification of synapses in the vertical “probes” became feasible. The constructed histogram of synaptic distribution (Fig. 9a) shows that synapses “form” one superficial synaptic stratum in the MZ and another deep, prominent stratum with several bins in the second CP–upper SP. The lower SP (former PSP) contains three times fewer synapses per 100 µm class (bin) than the upper SP. Four bins in the deep cortex (SP upper and SP lower) cover a relatively long cylinder of the cortical tissue. However, the complete histogram clearly shows the bilaminar distribution of synapses with the synapse-free cell-dense portion of the cortical plate (Fig. 9a).

**Fig. 10:**
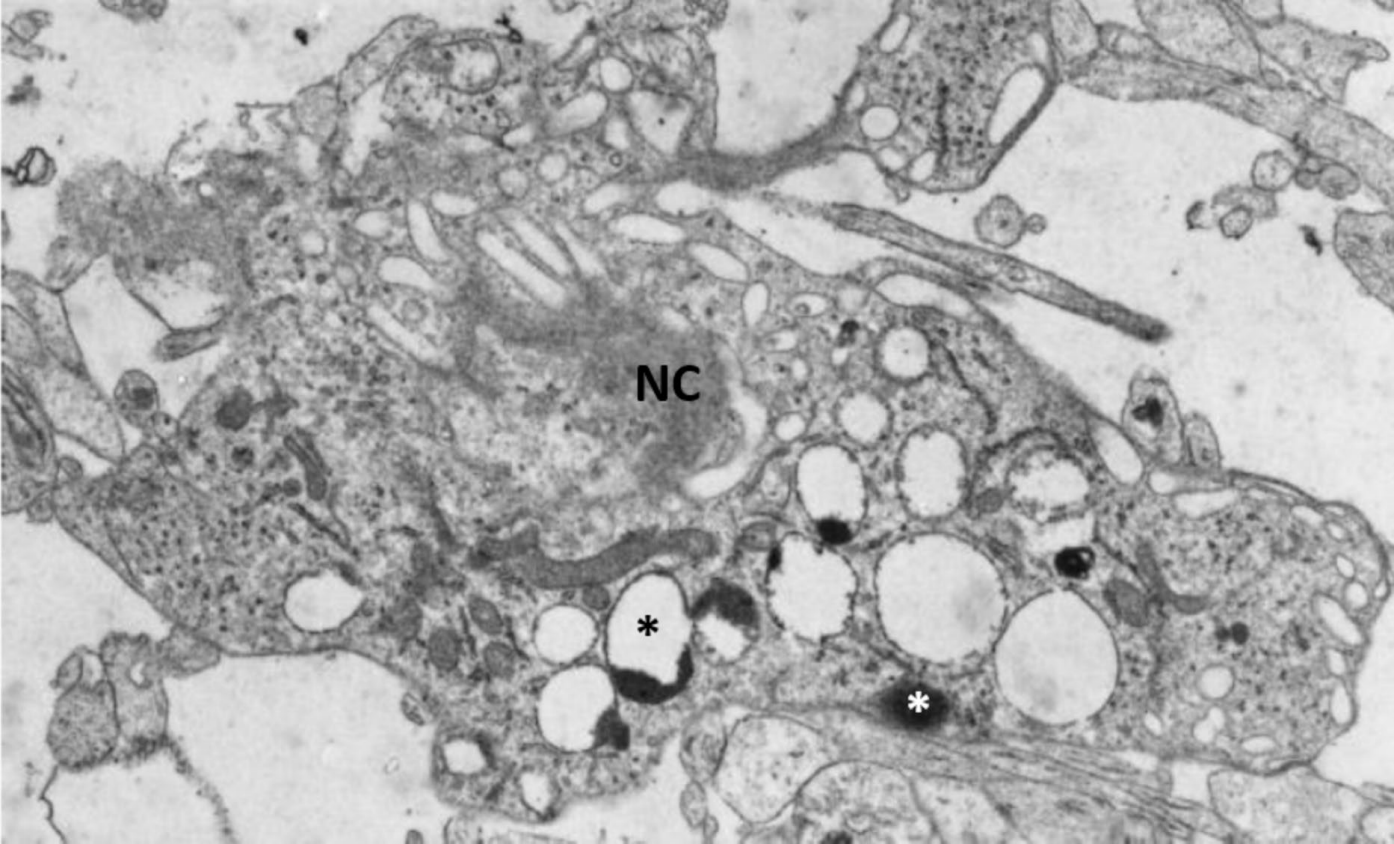
Apoptotic cell death encountered in the “second” plate during SP formation phase at 13 PCW. Note dark micronuclei (white asterisk) cup-like formations (black asterisk), swollen mitochondria and clustered chromatin (NC). Magnification 14,000×

The main feature of the deep, synapse-rich compartment (upper and lower SP) is the large amount of ECM, plexiform arrangement of small profiles of individual axons, smaller dendrites and polymorphic appearance of cells. A predominant number of synapses are of the axodendritic type (A1 and A2). However, there is a relatively high percentage of synapses on proximal dendrites, indicating that cells of the second plate and PSP neurons are definitively postsynaptic neurons. There is a relatively large number of intercellular junctions (puncta adherentia), especially in the marginal zone. In this phase, vertical probes revealed occasional “dying” neurons in the deep synaptic compartment (“second plate”). The cell death is apoptotic-like with micronuclei (Fig. 10, asterisk), cup-like formations (Fig. 10, asterisk), swollen or cystic mitochondria, clustered chromatin and relatively preserved cell membranes (Fig. 10).

SNAP-25 immunoreacted sections show a dense band in the marginal zone (Fig. 9a, arrow), non-reactive CP, and weak reactivity in the subplate compartment, whereas strong reactivity in the fiber bundles of the IZ (Fig. 9e). The strong reactivity in the MZ is predominantly in the tangential fibrillar component of the MZ. Immunoreactivity for ECM confirms the EM observation that synapse-rich compartments (SP and MZ) express stronger reactivity than the cell-dense cortical plate (Fig. 9f).

In vivo imaging of the fetal cerebrum during the subplate formation phase (Kostovic and Rakic 1990; Duque et al. 2016) is one of the most interesting findings of the present paper. In the lateral posterior coronal images at the level of the anterior hippocampal formation (Fig. 9b, arrowhead), all major cytoarchitectonic compartments of the cerebral wall except the marginal zone can be seen. The cell-dense cortical plate appears as a well-delineated band of low signal intensity. Leading deeper to the cell dense CP is the SP in formation, (second CP)–Fig. 9b between the red lines (SPf) of higher T2 signal intensity (lower cell density). These architectural landmarks are less discernible (Fig. 9b’) if the magnification scale is adjusted to a 1-µm Nissl-stained plastic section. However, cell dense portion of the cortical plate (Fig. 9b’) is a reliable landmark for bridging the scales between histology and in vivo MR imaging. The next compartment (towards the ventricle) is IZ with intermediate signal intensity followed by SVZ (deeper to red dotted line in Fig. 9a), end VZ along ventricular cavity showing increased cell density (lower signal intensity). Thus, a deep, synapse-rich compartment (upper and lower SP or second plate with pSP) was visible in the in vivo in utero imaging and delineable from the adjacent compartments (CP and IZ) on the basis of differential intermediate signal intensity.

### Secondary condensation of the cortical plate (phase of an enlarging subplate between 15–18 PCW)

**Fig. 11:**
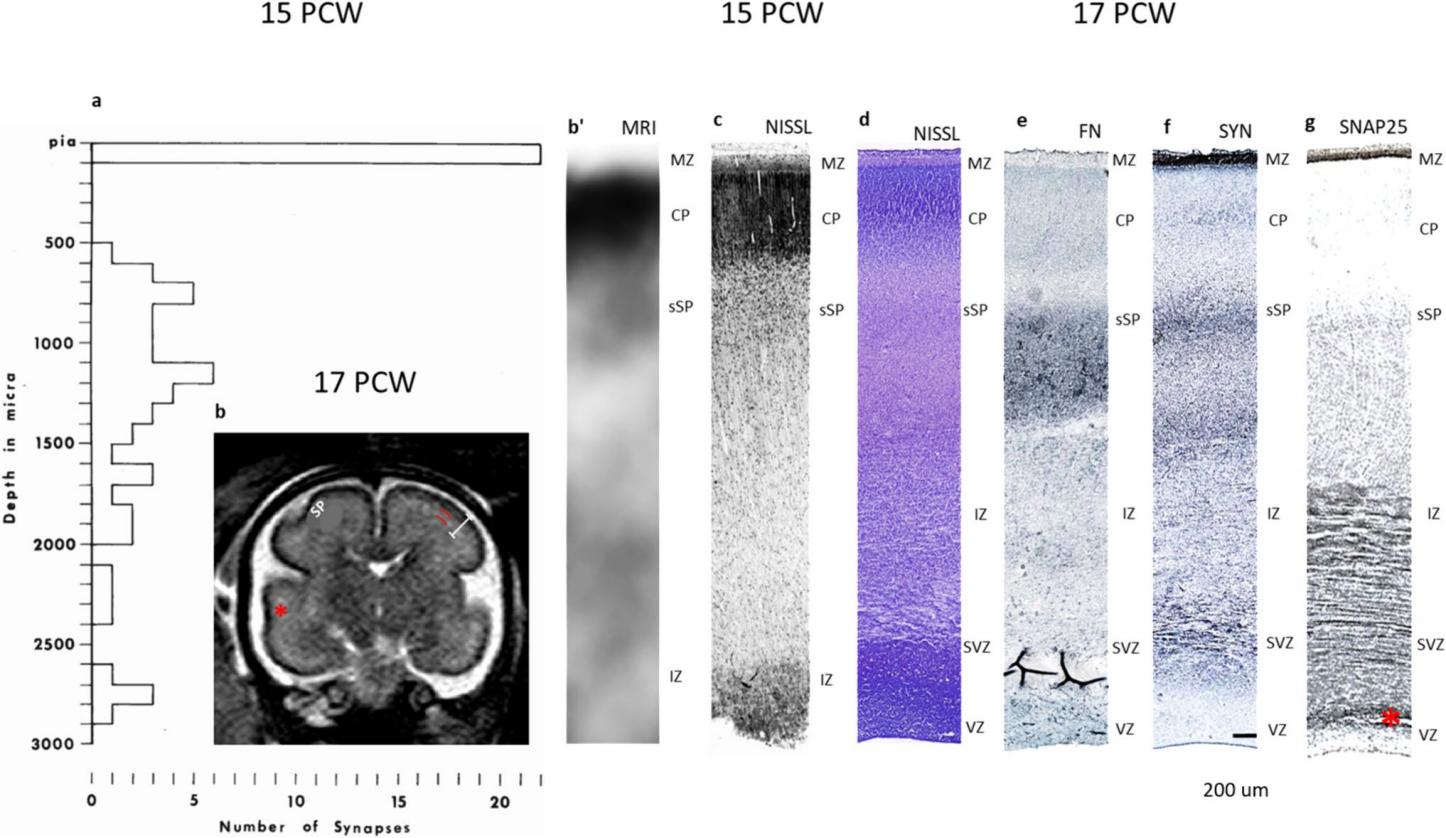
Bicompartmental distribution of synapses (**a**) during increase in SP volume (15–21 PCW) correlated with cytoarchitectonic laminar landmarks (**c**, **d**), expression of immunocytochemical markers (**e**–**g**) and in vivo MR laminar landmarks (**b** and **b’**). Histogram on (**a)** shows that density of synpses is highest in the MZ (the most superficial bin), no synapses were found in the CP while majority of synapses (bin from 500–3000 µm depth) was found in SP. On in vivo image (**b** low power inserted in histogram above apscissa) SP is delineated by 2 red lines and shows intermediate signal intensity: above SP is low signal intensity of CP and deep to the SP is IZ of somewhat lower signal intensity. SP is more voluminous in associative temporal cortex (asterisk) (**b**) high magnification of area depicted on **b** shows that below cell dense CP is thick T2 higher signal intensity (watery) zone (**b’**). On Nissl-stained 1 µm thick plastic section SP zone is approx 4 times thicker than CP and contains synapses throughout its thickness (**a** shows histogram of number of synapses found in 8 vertical probes). Synaptic marker synaptophysin is present throughout SP zone (**f**). Note that SNAP25 reactivity appears granular (**g**) in SP which is in constrast with fibrilat type of staining in SVZ. The callosal periventricular fiber rich zone (red asterisk on **g**) is also SNAP25 reactive. Pronounced delineation of SP is visible on section immunoreacted for extracellular marker fibronectin (**e**). **d**–**g** Show immunoreacted section from a single brain. Magnification on histogram **a** corresponds to magnification on **c** (plastic) and approx to the magnification of (**d**–**f)**. Area of inspected tissue is 144,000 µm^2^. Scale bar for IHC stainined tissue is 200 microns (**f**)

The cerebral wall is composed of the following cytoarchitectonic compartments:

The marginal zone shows sublaminar organization (Fig. 11c) and contains a superficial subpial granular layer, a middle, densely fibrillar sublayer and a thin “disrupted” sublayer at the interface with the cortical plate. The cell bodies of the large Cajal-Retzius cells are predominantly located in the superficial third of MZ; CP again shows high cell packing density (Fig. 11c, d) throughout its thickness (secondary “condensation” of the CP). Cell perikarya are arranged in well-pronounced, radially oriented embryonic cell columns.

Below the cortical plate is an enlarged subplate, which became the thickest cerebral compartment (approx. 2.5 mm). The subplate is characterized by low cell packing density and three sublaminae; superficial SP (Fig. 11, sSP) shows higher cell packing density than the intermediate portion of the subplate, the deepest part of the SP, forming the transitional more cellular sublamina towards the intermediate zone (Kostović et al. 2019a).

The intermediate zone is composed of fiber bundles, appearing as darkly “stained” on 1-micron thick sections and osmificated for EM analysis (IZ in Fig. 11c) as well as SNAP-25 immunoreacted fiber on the histological sections prepared for light microscopy (Fig. 11g).

The most significant changes observed in the 15 PCW old fetus are an increase in the overall number of synapses found in vertical probes of the cortex, the cortical plate does not contain synapses at any depth, the majority of synapses are found in the subplate, although the highest density of synapses is present in the MZ (Fig. 11a, histogram). It is notable that the superficial subplate contains a higher number of synapses per square area of inspected tissue than the deep subplate. A thick synaptic stratum is located in the superficial subplate compartment where there is a spatial overlap of dendritic branching of subplate neurons and dendrites originating from the deep cortical plate neurons (Mrzljak et al. 1988).

**Fig. 12:**
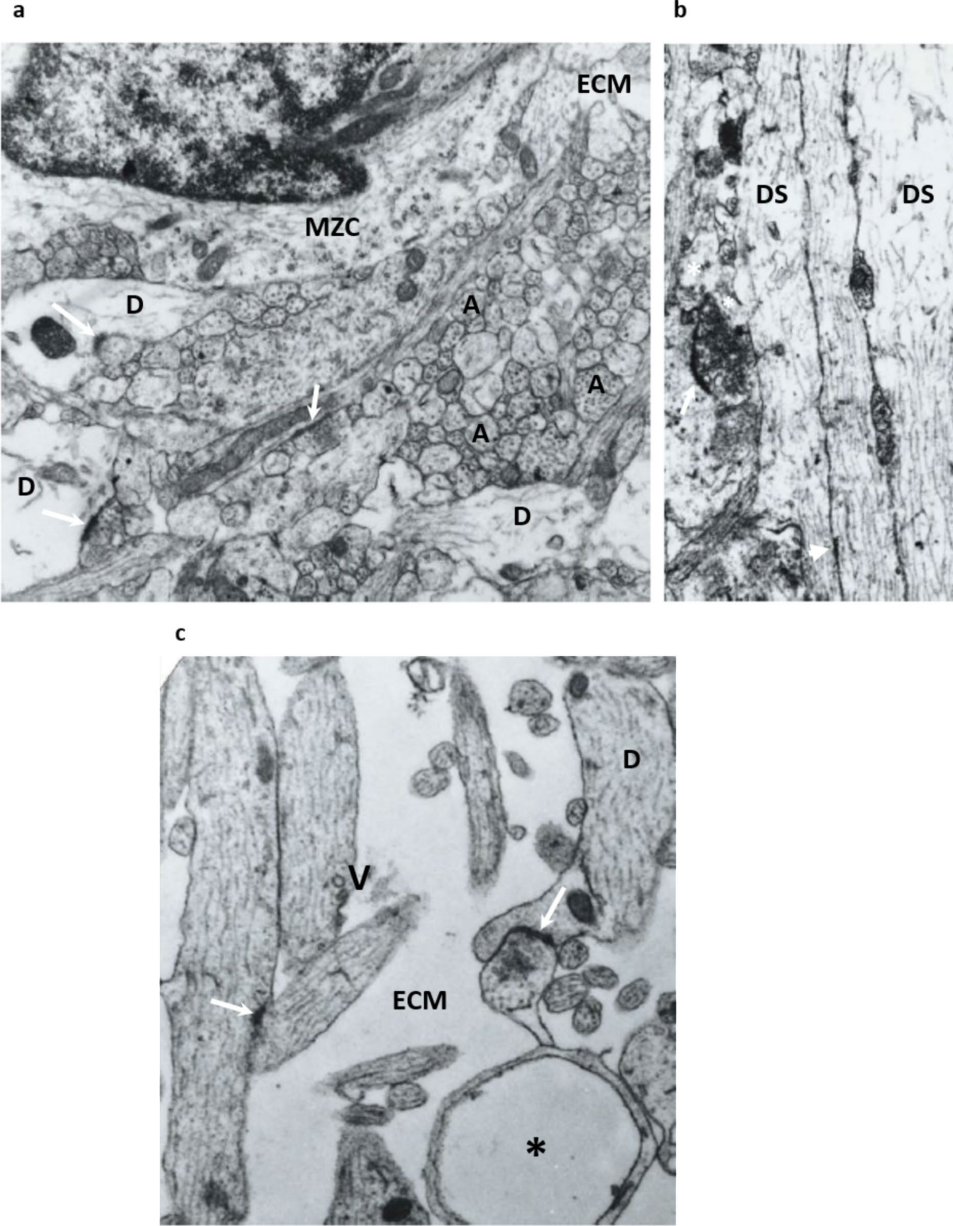
Typical ultrastructure of the neuropil in the MZ (**a**), CP (**b**) and SP (**c**). Densely packed non-myelinated axons (**a**) of small calibre which run parallel to the pial surface characterised by fibrillar organisation of the middle portion of marginal zone in a 15 PCW old specimen. Neuropil is more densely packed and higher number of synapses on apical dendrites (**d**) are visible throughout EM micrograph. Small cell (MZC) belong to the deepest portion of the subpial granular layer. ECM is relatively less abundant than in SP zone. **b** Shows neuropil of the CP in a 24 PCW old specimen. Note radially (vertically) oriented dendritic shafts (DS) of the pyramidal neurons which are densely packed displaying radial coherence of CP. Arrowhead indicates intercellular junction between 2 dendrites, while arrow indicates synapse on protrusion of dendrites (spin-like formation?). **c** Shows typical features of the SP neuropil: abundant ECM, plexiform arrangement of small calibre axons (some axons contain individual vesicles (V), and dendrites (**d**) of SP neurons. Asterisk is in the middle of circle-like “structure” which is bounded by close apposed cell membranes. Magnification on (**a**, **b**) 14,000x, on (**c**) 28,000x

The majority of synapses are formed between axon terminals and dendrites (types A1 and A2). The asymmetric synapses were seen occasionally on the cell bodies or proximal dendrites of the subplate neurons (types A4 and A5), indicating that SP neurons are postsynaptic neurons for fetal synapses (first described by Kostovic and Rakic 1990). The major feature of SP neuropil is the large amount of ECM (Fig. 12c, ECM) and numerous small, cross-sectional axonal and dendritic profiles (Fig. 12c). In the neuropil, circular membrane-bounded structures can be identified (Fig. 12c, asterisk), but our search did not find a definitive engulfment of synapses by microglia or astroglia. Some axons contain individual vesicles (V in Fig. 12c), indicating trafficking along axons. These ultrastructural features prove that the subplate neuropil is characterized by a plexiform arrangement of various cellular elements that are embedded in rich ECM. In contrast to the subplate, the neuropil of the marginal zone is more densely packed with small cell profiles and synapses, showing less abundant ECM and numerous small bundles of axons (Fig. 12a).

Nissl-stained 1-micron thick plastic sections (Fig. 11c), serving as a basic spatial reference for the histogram of laminar distribution of synapses (Fig. 11c), were obtained from specimen 15 PCW. Immunocytochemically treated sections belonging to a 2-week older specimen (17 PCW) and included in the analysis to match in vivo MR image of the 17 PCW old fetus (Fig. 11b). The comparison between the immunocytochemical pattern of lamination and synaptic distribution was successfully performed for the marginal zone and cortical plate only, while the exact correlation with deep borders of the subplate compartment is difficult due to the different depth scale of shrunken paraffin-embedded tissue. In the marginal zone, strong synaptophysin and SNAP25 reactivity corresponds to the synapse-rich stratum on the histogram (Fig. 11a, f). The synapse-free CP shows weak synaptophysin reactivity except for some fibrillar staining in the middle of the cortical plate. Regarding the subplate compartment, a good correlation between synaptophysin and fibronectin reactivity and synaptic stratum is evident for the superficial subplate only (Fig. 11a, c, e, h). However, Nissl-stained paraffin sections (Fig. 11d) showing a deep border of the cortical plate are good landmarks for comparing different immunostained paraffin sections with shrunken SP and non-shrunken SP compartments on 1-micron plastic section. In line with the previous phase of development, axon-rich fibrillar layers in IZ and SVZ (periventricular, callosal, fiber-rich zone – asterisk in Fig. 11g) display synaptophysin and SNAP25 immunoreactivity. SNAP25 immunoreactivity appears granular in SP (Fig. 11g). This is in contrast fibrillary type of staining in SVZ and callosal periventricular fiber rich zone (red asterisk on Fig. 11g) which divides SVZ in two sublaminae.

The subplate compartment is defined as an irregularly delineated zone of the intermediate signal intensity (between lines of the bar in Fig. 11b) situated between a low-signal, cell-dense cortical plate (Fig. 11b’) and the intermediate zone of a somewhat lower signal intensity. In the temporal lobe (Fig. 11b, asterisk), the subplate shows better delineation than in the somatosensory cortex due to the different geometry of fibers. However, the precise delineation of the subplate on a Nissl-stained 1-micron thick plastic section (Fig. 11c) is the most reliable because all fibers, regardless of orientation, show dark staining due to osmification providing precise vertical orientation of EM blocks.

### Stationary subplate phase—first lamination within the cortical plate (22–26 PCW)

**Fig. 13:**
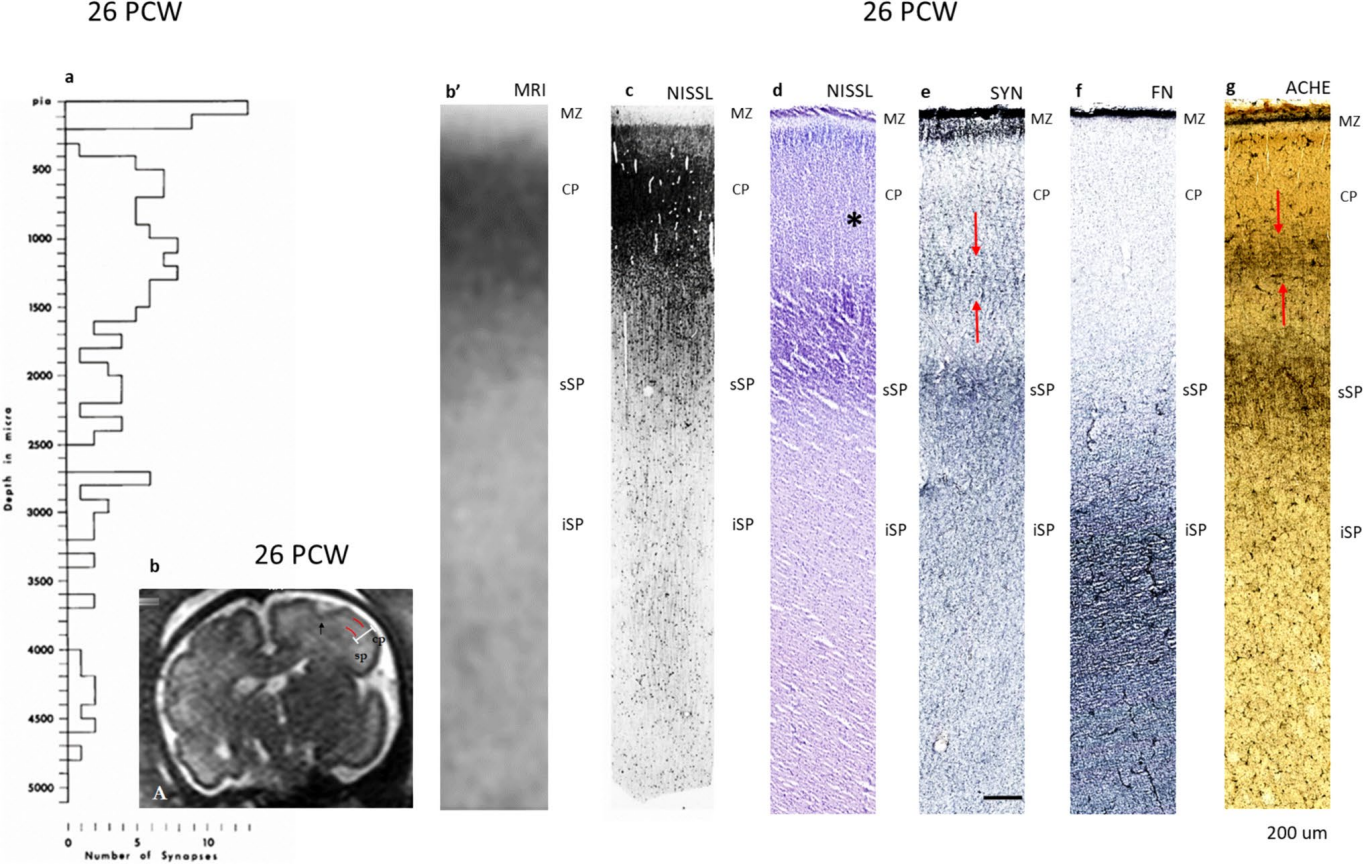
Three-compartmental synaptic distribution (MZ, CP, SP) is shown on histogram (**a**) of a 26 PCW analyzed specimen. This pattern of synaptic distribution begins after onset of synaptogenesis in the CP and was observed in specimens between 22 and 26 PCW (specimens between 15 and 22 PCW were not analyzed quantitatively). SP is still the most voluminous synaptic compartment and contains synapses throughout its whole thickness. Density of synapses of “3rd” compartment in the CP is higher than in the SP (“ascending” synaptogenesis). Corresponding laminar (compartmental) correlates are shown in in vivo images (**b** and **b’**) and histological sections stained or reacted with different cellular, synaptic and fibrillar markers (**c**–**g**). **d**–**f** Show immunoreacted section from a single brain. Note synaptophysin rich band in the middle of the CP and simliar band on AchE preparation (between arrows). On in vivo image (**b** low power inserted in to the histogram space above apscisse) the size of the SP band of higher T2 signal intensity is visible also on high magnification (**b’**). Magnification scale applies also for Nissl stained one micron Nissl section (**c**) and cortical layers (MZ, CP, SP) are alligned with histological sections shown on (**d**–**g)**. SP is delineated by red lines and is characterized by high signal intensity on T2 sequence. Arrow indicates formation of primary sulci with corresponding reduction of SP thickness. Area of inspected tissue is 240,000 µm^2^. Scale bar for IHC stainined tissue is 200 microns (**e**)

The cerebral wall is composed of the same laminar compartments as during the previous phase. The subplate has a greater thickness and extends beyond the cortical depths which can be “covered” by a single 1-micron thick plastic section or adjacent 5-mm-long ultrathin section (Fig. 13c). The overall low cell packing density is the main cytoarchitectonic feature of the subplate compartment, which is best pronounced in its intermediate sublamina (Fig. 13c, d), while the superficial subplate (Fig. 13, sSP) shows higher cell packing density. The vertically precisely oriented 1-micron thick plastic sections show great variability of cell sizes and morphologies. The fine radially oriented fibers stained with osmium are also visible. During this developmental phase, the first lamination in the middle of the cortical plate was seen. This lamination is hardly discernible on 1-micron thick plastic sections when compared to thicker Nissl-stained paraffin sections (Fig. 13d, asterisk). For a detailed description of the sublaminar organization of subplates, see Kostović et al. (2019a).

During this phase, there are three synapse-rich compartments (a three-compartmental pattern), as shown in Fig. 13a. These three synapse-rich compartments are the marginal zone (MZ), the deeper 2/3 of the cortical plate (CP) and the subplate (SP). The marginal zone shows two strata. The superficial half of the marginal zone has a somewhat higher synapse density than the deep portion of the MZ (Fig. 13a).

At this age, the first synapses appear within the deep portion of the cortical plate, as found in the youngest specimen examined in this developmental group (at 22 PCW—not counted). At 26 PCW, synapses occupied the 2/3 deep of the cortical plate and showed two groups of synaptic strata in the middle of the cortical plate. The highest synaptic density is in the middle portion of the cortical plate and the second group is at the interface between the cortical plate and sSP (Fig. 13a). Within the subplate, there are several strata of moderate synapse density intermingled with strata with no synapses (Fig. 13a), indicating dispersion of circuitry elements in this compartment. The pattern of synaptic development from deep levels (the interface between superficial subplate and deep cortical plate) towards the most superficial, cell-dense cortical plate can be described as “ascending” synaptogenesis.

In this synaptogenesis phase, asymmetric synapses between axon terminals and dendrites (type A1 and A2) also represent a vast majority of synaptic junctions. Within the SP, synaptic junctions can be readily found on proximal dendrites of the SP neurons (type A4). The presence of synapses on vertically oriented, thick dendritic shafts (DS on Fig. 12b) is a distinguishable feature of the cortical plate synapses (type A7). There is an increase in the number of double synaptic contacts in both the marginal zone and cortical plate (Fig. 1f). For the first time in development, postsynaptic profiles occasionally protrude from dendritic shafts resembling spine-like formations (type A10 in Fig. 12b). The major change in synaptic neuropil occurs in the cortical plate where radially oriented cell processes are densely packed together, forming bundles of vertically aligned dendritic shafts, which is a characteristic of this synaptic compartment (Fig. 11b). The middle portion of the MZ is crowded with bundles of small size axons that run parallel to the pia surface. The MZ also contains characteristic non-synaptic intercellular junctions with long apposition of membrane-associated density (J3 on Fig. 3b).

The most significant finding is that synaptophysin immunoreactivity corresponds closely to the synaptic distribution, as revealed on the histograms. Two bands are visible in the MZ, in the middle of the CP there is a immunoreactivity band (Fig. 13d, between the arrows), corresponding to the highest density of synapses, as revealed in the histogram in Fig. 13a. Synaptophysin immunoreactivity is distributed throughout the subplate with a higher concentration in the sSP. This laminar pattern with the band in the CP (Fig. 13g, between the arrows) is also obvious in histochemically treated sections for AchE.

Notable in the in vivo images is a “watery” high T2 signal intensity, corresponding to the synapse-rich subplate compartment that has increased in thickness (Fig. 13b, SP, between lines of the white bar). The CP is sharply delineated (CP on Fig. 13b) and is approximately 3–4 times thinner than the subplate. The delineation of the deep border of the SP is less pronounced and irregular. The ratio of the subplate: CP thickness in in vivo images (Fig. 13b’) corresponds to the ratio visible on Nissl-stained 1-micron plastic sections (Fig. 13c). Subplate thickness is reduced at the sites of the formation of primary sulci (Fig. 13b, arrow). This reduction of the subplate at the points of sulci formation corresponds to the finding presented by Kostović et al. 2014. In the associative cortical regions (temporal, parietal) where primary cortical gyri increase in size (Fig. 13b, Sp), the subplate compartment is more prominent and appears as a T2 high signal “balloon” in the core of the primary gyri.

## Discussion

By coupling different scales of resolution (EM, light microscopy, in vivo MR) we have shown that laminar compartmental distribution of synapses was closely related to the cytoarchitectonic and synaptic immunocytochemical landmarks at a light microscopical level and successfully transposed to in vivo in utero MR images of the early human somatosensory cortex. For the first time, we have shown developmental changes of synapse containing compartments throughout the whole midgestation period (from 8 to 26 PCW), and demonstrated that bilaminar distribution of synapses (SP and MZ) is maintained even during primate characteristic expansion of the SP around 13 PCW (Duque et al. 2016) when quantifiable number of synapses was found in the cell loose “second” plate. Furthermore, we have identified three-compartmental (SP, CP, MZ) phase of synapse distribution around 22 PCW when synapse production proceeds from the deep to the middle CP (“ascending” synaptogenesis). These results show prolonged process of formation of synapses in the human fetal cortex which corresponds to the gradual and sequential ingrowth of preterminal axons above and below CP. Selective presence of synapses in two transient synaptic compartments indicates unique properties of their neuropil, where plexuses of small caliber axons and their growth cones meet postsynaptic elements of SP and MZ neurons and basal and apical dendrites of early born pyramidal cells of CP. These postsynaptic elements are embedded in the abundant ECM (Charron and Tessier-Lavigne 2005; Jovanov-Milošević et al. 2014) rich in morphogens, guidance cues, and membrane active molecules. The radially oriented pyramidal cells of the CP which are surrounded by tangential synapse rich SP and MZ compartments outline the fundamental neuronal network architecture in the human fetal cortex (Marin-Padilla 1971; Kostović-Knežević et al 1978; Mrzljak et al. 1988; Meyer et al. 2000; Aboitiz and Montiel 2007; Bystron et al. 2008). How this structural and developmental aspects of early compartmental distribution of synapses in the human fetal cortex may explain morphogenetic and possible functional role of pioneering synapses will be elaborated in the following paragrahs of discussion.

### Structural characterization of early cortical synapses and their pioneering role in the development of functional cortical circuitry

The formation of a quantifiable number of structurally well-defined synapses (Molliver et al. 1973; present data) in the early human fetal neocortex within the transient cell compartments (Kostovic and Rakic 1990; Kostović 2020) is an important neurogenetic event which sheds light on the early spatiotemporal framework for the structural and functional development of complex human cortical networks. Based on the results of the present study, it is obvious that first synapses engage early-born pioneer neurons of the marginal zone and subplate (Marin-Padilla 1971; Kostovic and Rakic 1990; Meyer et al. 2000; Bystron et al. 2008; Molnár et al. 2019) and also dendrites of first-born deep pyramidal neurons (Marin-Padilla 1971; Kostović-Knežević et al. 1978). Thus, it seems appropriate to call the first synapses the “pioneering” synapses because they connect pioneering neurons of the developing fetal cortex. It should be noted that early synapse formation requires precise developmental orchestration of transcriptional and posttranscriptional programs which are already established at that period of prenatal development (Kwan et al. 2008; Hoerder-Suabedissen and Molnár 2015; Polioudakis et al. 2019) and show a significant expression of synapses-related gene sets (Wagstyl et al. 2024). Displaying some novel human-specific characterization (Luria et al. 2023).

For proper evaluation of neurodevelopmental significance and the prospective morphogenetic role of “pioneering” fetal cortical synapses, several questions should be answered. In the following text we will answer these questions and analyze answers in separate paragraphs with subheadings.

1. Do fetal synapses meet all ultra-structural criteria defined by previous electron microscopy studies to be considered specific synaptic junctions (Peters et al. 1970)?
  The data presented in the present paper clearly provides an answer to this question by showing that all synaptic junctions we have used in analyzing spatial distribution, regardless of the combination of the morphology of presynaptic and postsynaptic elements (our types A1–A10, type S synapses) show ultrastructural features used to define chemical synapses: presynaptic and postsynaptic membrane associated densities, synaptic cleft, and synaptic vesicles clustered in the presynaptic element (Colonnier 1968; Peters et al. 1970). Most of the excitatory synapses are probably glutamatergic and asymmetric (Grey 1 type) (Colonnier 1968; Peters et al. 1970; DeFelipe et al. 1999). We observed symmetrical junctions in 10% of all synapses, which were predominantly located on cell soma or proximal dendrites. These types of symmetrical synapses are traditionally considered GABAergic ( Peters et al. 1970; DeFelipe et al. 1999). However, in the developing cortex GABA is a depolarizing neurotransmitter during early phases of corticogenesis (Ben-Ari et al. 2012). The switch to GABA hyperpolarizing responses related to maturation of the KCC2 molecular system was observed as early as 16–18 PCW (Bayatti et al. 2008; Sedmak et al. 2016).
2. How are observed typical synapses engaged in the transient spontaneous or endogenous circuitry?
  This question concerns the role of pioneering synapses in the transient spontaneous (endogenous) activity of the fetal cortex. At first glance, our finding of the predominance of well-defined asymmetric synapses seems to be contradictory to experimental findings where electrical contacts (gap junctions) and not conventional synapses drive early spontaneous cortical activity in the developing rodent brain (Yuste et al. 1992; Hanganu et al. 2001; Luhmann 2022;) and in vitro studies on human material (Moore et al. 2011).
  Gap junctions are also important for non-synaptic communication of spontaneously co-active neurons during the postnatal formation of neuronal domains (Yuste et al. 1992). Gap junctions are composed of hemichannels connexin (Fukuda 2007). However, despite systematic analysis of all cortical synapse-rich compartments in the present study, we did not find gap junctions between neuronal cells that would meet the criteria described for “classical” electrical synapse (Peters et al. 1970; Fukuda 2007). In contrast, we found numerous cell-to-cell junctions of various lengths and topography that meet the criteria for adherence junction (Peters et al. 1970). They seem to be more frequent in younger specimens. According to Connors and Long 2004 “gap junctions are notoriously difficult to observe, and absence of gap junctions cannot be considered as evidence for the absence of electrical coupling.” These authors point to the findings of Williams and DeHaan 1981, who claim that gap junction channels may be functional even when they are too widely dispersed to form conventional gap junctions. It is our opinion (Kostović 2020) that there are additional possibilities that may explain discrepancies between our failure to find typical gap junctions and experiments on brain slices showing an electrical type of oscillatory synchronized networks. First, physiologically well-defined developmental connexin hemichannel-driven networks may have different ultrastructural correlates than typical gap junctions.
  Second, thinner brain slices prepared within an hour of receiving the fetal samples (14–17 PCW) show colocalization of pre- and post-synaptic elements in SP by confocal microscopy, for both GABAergic and glutamatergic synapses displaying evoked neurotransmitter release and spontaneous glutamatergic and GABAergic currents in SP neurons (McLeod et al. 2023). This new data obtained on organotypic cultures of the human SP (McLeod et al. 2023) and data presented in the present paper on abundance of typical synapses in SP, together with our failure to localize “classical” electrical synapses, suggest that chemical synaptic transmission after 13 PCW becomes an important driver of spontaneous SP activity in the human fetal cortex.
  However, in the early phases of the development of experimental models and very early preplate of the human telencephalon where there are no synapses, the most plausible explanation for early endogenous activity is found in a paper by Kandler and Katz 1995: “communication via gap junctions may generate coordinated electrical or biochemical activity before the onset of synaptic transmission.”
  Furthermore, on the basis of our results, we cannot answer whether the differentiation of synaptic morphology also modifies synaptic transmission (Couteaux 1963). In addition, we cannot exclude silent synapses solely on the basis of EM criteria (Isaac et al. 1997).
3. What are other prospective cellular sources of transmitter release?
  In the slowly developing human fetal cortex, in contrast to the rodent cortex (Bayer and Altman 1990; Molnár and Clowry 2012; Marx et al. 2017; Molnár et al. 2019; Alzu’bi et al. 2019), there is a prolonged overlap of different, developmentally specific cellular mechanisms, leading to a mixture of hemichannels, neural transmission through non-synaptic mechanisms such as transmitter released from growth cones (Young and Poo 1983), paracrine release of active substances (Molnár et al. 2020), extrasynaptic release of transmitters (Sesack et al. 2003), volume transmission (Descarries and Mechawar 2000), ion communication through ECM (Teleman et al. 2001), as well as typical chemical transmission (Molliver 1982; Kostović 2020). Therefore, in the protracted, gradual, step-by-step development of the human neocortex during the first several fetal months, there may be a prolonged transient period of co-existence and interaction (Pereda 2014) of electrical coupling (Molnár et al. 2020; Luhmann 2022), and various types of chemical transmission (Molliver 1982). The exact proportion of the different sources of excitability and generation of electrical response can be complicated, as stated earlier, due to the paracrine mechanisms of transmitter release. In fact, the extraordinarily rich plexus of fast-growing preterminal and small axon branching (Portera-Cailliau et al. 2005; Kalil et al. 2011; Rockland 2020) in close proximity with adherent membranes of “postsynaptic” neurons may be a hitherto unrecognized source of release of membrane-active (transmitters) substances and substrate of cortical activity in the developing cortex.
  Axonal transport and intraaxonal synthesis of t-SNARE proteins (Batista et al. 2017) requires continuated balance which may control spatial distribution of synapses (Jin and Garner 2008; Wu et al. 2013). The presence of strong reactivity for synaptic markers of cortical afferent fibers on formalin fixed material of histological sections (Chun and Shatz 1988; Bayatti et al. 2008; Harkin et al. 2017; Žunić Išasegi et al. 2018; Kostović et al. 2019a; Junaković et al. 2023; Sarnat 2023) supports importance of axonal transport and intraaxonal synthesis of synaptic proteins during cortical development. However, this type of expression of synaptic markers (Eastwood et al. 1994; Südhof 2012; Vadisiute et al. 2022) seems to be less expressed in synaptic strata which may be best revealed with systematic EM analysis.
4. When during fetal life, do ultrastructurally defined “classical” synapses begin to express functional properties of chemical synapses, that is, release transmitters and undergo subsequent changes in excitability of the postsynaptic membrane (Couteaux 1963; Molnár et al. 2020), and participate in the permanent thalamocortical circuitry?
  Concerning the question of when chemical synapses gradually replace early, spontaneous, predominantly electrical coupling (Molnár et al. 2020; Kostović 2020; Luhmann 2022), we propose that after 22 PCW, when thalamocortical synapses are being rapidly produced on the dendrites of the cortical plate, the critical period (three-compartmental pattern, “ascending” synaptogenesis) begins when chemical synapses start to dominate activity in the human fetal cortex. During this period, thalamocortical fibers relocate to the CP and contact both neurons within the CP and the SP neurons (Kostović 2020).
  This period corresponds to prematurely born infants, which is clinically extremely important due to the establishment of thalamocortical circuitry (Kostović and Judaš 2010; Vanhatalo and Kaila 2010; Karolis et al. 2023; Taymourtash et al. 2023; Wilson et al. 2023; Zheng et al. 2023). An ascending increase in synapses in the superficial cortical plate may explain the appearance of surface negative cortical responses in the late prenatal period (Molliver and Loos 1970).
  Remarkably, in the period after 22 PCW, a massive relocation and “invasion” of the thalamocortical axons into the cortical plate occurs (Kostović and Judaš 2010; Kostovic and Goldman-Rakic 1983; Kostovic and Rakic 1984; Krsnik et al. 2017) together with the differentiation of dendrites within the cortical plate and development of local circuitry neurons (Marin-Padilla 1971; Mrzljak et al. 1988). The thalamocortical connectivity was visualized in current DTI tractography studies (Huang et al. 2009; Vasung et al. 2017; Karolis et al. 2023) and comprehensively elaborated in a recent paper by Wilson et al. 2023. In the recent study of Taymourtash et al. 2023, a time window for a peak increase of thalamocortical functional connectivity intensity was found to correspond to the invasion of thalamocortical axons and associated synaptic engagement in the cortical plate.
  During the period associated with prematurely born infants (after 22 PCW–the three-compartmental phase), thalamocortical and intracortical synapses became functional for both spontaneous activity as recorded by the classical EEG (Dreyfus-Brisac and Larroche 1971; Vanhatalo and Kaila 2010), the appearance of spontaneous transient activity (Kidokoro 2021), cross-frequency coupling, as well as evoked functional responses as revealed by various stimulation and recording techniques (Fitzgerald 2005; Mahmoudzadeh et al. 2013).
  It was shown that early functional connectivity between the thalamus and sensory cortical areas has paramount significance for the proper establishment of sensory cortical networks (Van Der Loos and Woolsey 1973; Rakic 1977; Friauf et al. 1990; O’Leary et al. 1994; Katz and Shatz 1996; Kanold and Luhmann 2010; Colonnese and Phillips 2018; Henschke et al. 2018; Cadwell et al. 2019; Molnár et al. 2020; Taymourtash et al. 2023; Wilson et al. 2023). The establishment of thalamocortical circuitry in preterm infants implies transience from the sensory expected to sensory evoked phases of cortical development with an increasing influence of environmental stimuli, although the actual number of synapses does not necessarily change (Bourgeois et al. 1989). Multiple roles of early thalamocortical acticivity were studied in different experimental species with sophisticated physiological methods (Molnár et al. 2003; Higashi et al. 2002, 2005; Molnár and Kwan 2024). Recent observations in macaque and human revealed that thalamic projections also interact with some of the cortical progenitor populations in the germinal zones (Molnár et al. 2023).
  Given that thalamocortical axons innervate the cortex in a topographically organized manner, they enable sensory input to refine cortical arealization (Molnár and Kwan 2024). Thus, pioneering synapses of this three-compartmental phase pave the way for establishing functional sensory networks and the refinement of the functional areas and neuronal maps in the sensory cortex. They can also activate genes that regulate developmental molecular mechanisms and functional maturation in the postsynaptic neurons of the developing cortex (Armstrong and Montminy 1993). This process is particularly important in the developing human cortex, where there is a prolonged co-existence of transient SP and permanent cortical circuitry (Kostović 2020).
5. What proportion of fetal synapses are transient, and which synapses will survive and participate later in life within the permanent cortical circuitry (Rakic et al 1986; Kostovic and Rakic 1990; Kostović, 2020; Molnár et al 2020)?
  One may argue that these “transient” synapses will be later pruned because the final circuitry is not yet established (Rakic et al. 1986; Huttenlocher and Dabholkar 1997; Petanjek et al. 2011). Postnatal pruning happens mostly with synapses on spines (Petanjek et al. 2011) while in the prenatal cortex during the period covered in the present study, where there are very few spines on pyramidal and non-pyramidal neurons (Mrzljak et al. 1988), prunning of synapses on spines is not very likely. In the perinatal cortex, a prospective reduction of synapses in the subplate can also be caused by the retraction of exuberant axons, which is expected in humans after 28 PCW (Innocenti 2020; LaMantia and Rakic 1990). The fate of some synapses may depend on the fate of postsynaptic SP and MZ neurons. So far, evidence obtained in human (Kostovic and Rakic 1980) and monkey (Kostovic and Rakic 1980; Ahmed et al. 2024) shows that a great number of SP neurons survives as interstitial neurons of the white matter and these neurons have typical synaptic contacts (Kostovic and Rakic 1980). Despite regional variations in the number of interstitial neurons there is a consistent correlation between number of interstitial white matter neurons and the number of layer V and VI neurons studied (Ahmed et al. 2024).
  Finally, we should be aware that after 22 PCW, the excitability of synaptic membranes can be changed by astroglial activity (Verkhratsky and Parpura 2015). Astrogliogenesis begins very early in the fetal primate cortex (Kostović et al 2019a; Schmechel and Rakic 1979), and astrocytes can release various chemical transmitters, i.e., “gliotransmission”, and participate in bidirectional signaling with neurons (Verkhratsky and Parpura 2015). Our results on early synaptogenesis in the human fetal cortex can explain, to some extent, the early appearance of lamina-characteristic types of astroglia as prospective players in synapse formation of the human fetal cortex (Kostović et al. 2019a). Besides molecular interactions and signaling at synaptic sites, astrocytes can synthesize ECM molecules and provide a substrate for axonal guidance and recognition of postsynaptic neurons. This early fetal appearance of astrocytes in the primate prenatal cortex is different from rodent models, where astrogliogenesis is predominantly a postnatal event. The early appearance of astrocytes can particularly reinforce the chemical transmission of glutamatergic synapses (Eroglu and Barres 2010). Thus, astrocytes seem to be underestimated players in the synaptogenesis of the cerebral cortex in fetuses and preterm infants.

### The significance of visualization of synapse-rich compartments in the early human fetal cortex for understanding human intrauterine life and origin of neurodevelopmental disorders

From a biological point of view, early fetal “pioneering “synapses are initial points of neuronal interactions necessary for the establishment of postnatal functional networks (Katz and Shatz 1996; Sporns et al. 2004; Fair et al. 2008; Fransson et al. 2011; Schöpf et al. 2012; Gao et al. 2015; Turk et al. 2019; Molnár, et al. 2020). Therefore, in vivo visualization of synapse rich compartments is likely to receive more attention in the future studies of normal and altered development of human cortex. It is proposed that fetal synapses are involved in the intrauterine motor behavior (Prechtl and Hopkins 1986; Hadders-Algra 2018; Kurjak et al. 2019) and may underlie early resting-state functional activity in utero (Schöpf et al. 2012; Thomason et al. 2017).

Besides the neurobiological significance of prenatal synaptogenesis in the establishment of interneuronal connectivity, the early development of synapses in the human fetus is important for understanding specific aspects of human prenatal life and ethical questions concerning fetal pain and abortion policies. From a clinical aspect, understanding the significance of thalamocortical synapses (Kostović and Judaš 2010) is important for interpretation of cortical response to pain stimuli in early preterm infants (Fitzgerald 2005; Lee et al. 2005). However, despite the permanent interest in the synaptogenesis of the human cortex, their role in pain perception, fetal consciousness and pathogenesis of mental disorders is far from an acceptable explanation. The fetal pain issue is still under debate. The predominant opinion is that after 22–26 PCW, thalamocortical synapses can be responsible for pain reactions (Fitzgerald 2005; Thill 2023), although the nature of cortical pain processing in the human fetal cerebrum remains obscure (Thill 2023). Before the establishment of thalamocortical connectivity, the capacity for pain response seems to be significantly reduced. However, the “subplate modulation hypothesis” (Thill 2023) indicates that a well-developed subplate nexus (Kostović 2020) is sufficient to facilitate pre-conscious pain response in human fetuses.

The prospective involvement of fetal synapses in behavioral and “preconscious” experience of the human fetus is complicated by the issue of the poorly understood real state of fetal consciousness (Lagercrantz and Changeux 2009; Schore 2015; Ferber et al. 2023). One hypothetical view is that during the late fetal-preterm period, the poorly understood “preconscious” state emerges (Lagercrantz and Changeux 2009) due to the establishment of the somatosensory thalamocortical system, which plays an important role in awareness of one’s own body.

Our study offers, as a first step, to bridge the scales of resolution and monitor synapse-rich compartments at a macroscopic level of in vivo imaging of the fetal and early preterm cerebrum. At the moment, this approach is ethically suitable only after 22 PCW. Thus, failure to find proper laminar organization of the cerebral wall with the presence of characteristic signal intensity within the subplate may serve as an indirect, coarse spatial marker of abnormal synaptic development. However, changes to the signal and volume of a subplate may serve as a starting point for more precise pathogenetic studies. Unfortunately, the other synapse-rich compartment in the marginal zone cannot be visualized using current MR technology. For the visualization of the third synapse-rich compartment within the CP (after 22 PCW), it is crucial to analyze changes in the radial coherence (McKinstry et al. 2002), cortical isotropy and the appearance of lamination in the CP (Kostović and Judaš 2010) which can be correlated with ascending synaptogenesis as described in the present paper. Our results show that intermediate signal intensity of SP situated between cell-dense CP and fibrillar IZ (fetal white matter) is coarse but constant marker of synapse rich compartment giving the new insight into organization of micro circuitry of the fetal cortex. The small number of synapses within synapse-rich compartments does not underplay the significance of fetal synapses and the need to analyze synapse-rich compartments at different scales of magnification. Namely, it is our opinion that the first fetal synapses play crucial pioneering developmental, functional and prospective inductive morphogenetic roles in the coupling of early subcortical inputs (modulatory, thalamic, cortical) with crucial postsynaptic elements on subplate neurons and dendrites of pyramidal neurons of the cortical plate (Kostović 2020). All of the early functional synapses are very likely important for maintaining transient connectivity, the survival of neurons and strengthening cortical input–output functions. This possibility complies with the well-known role of functional activity in the development of neural networks (Friauf et al. 1990). The presence of the subplate and cortical plate and transient synaptic associative networks in the late fetus also conforms to evidence where the landscape of brain connectivity is already established by the time of birth in the human brain (Huang et al. 2009; Kostović et al. 2019a).

Regarding hypoxic damage in the developing brain, it is well known that during late phases and near term there is an increased vulnerability of so-called “grey matter”, which is different from early vulnerability affecting most of the “white matter” during early preterm age (Volpe 2012). These findings may imply that in early preterms, lesions of fetal white matter rather than damage to synapse-rich compartments dominate in hypoxic-ischemic lesions. However, previous findings suggest that the subplate as synapse-rich compartment may also become lesioned in hypoxia–ischemia (Kostović et al. 2014).

The well-known mechanisms of neuroexcitotoxicity due to excessive glutamate release in hypoxia–ischemia (Volpe 2012) are in line with the precocious maturity of glutamatergic neurons in the subplate compartment. The existence of synapses is an essential pre-requisite for interpreting the effect of hypoxia-inducible factor signaling mechanism in CNS and consecutive failure of synaptic transmission following hypoxia (Corcoran and O’Connor 2013). Recent studies have pointed to the neurosecretory role of SP neurons because these neurons were identified as the main source of neuroserpin (Adorjan et al. 2019). This SP function reveals a novel potential molecular modulation of hypoxic-ischaemic injury (Millar et al. 2017). Recently it was also shown that SP neurosecretory neurons have highly developed rough endoplasmatic reticulum (ER) which changes under stress conditions (Kondo et al. 2015). This finding shows potential of electron microscopy as a starting level in a multi-scale approach. Our EM images show well developed ER in both SP and Cajal-Retzius neurons of the MZ. However, we cannot find typical ER stress changes due to the fact that our EM specimens are presumably “normal”.

In conclusion, the results in the present paper suggest that the study of damage to the synapse-rich compartment subplate and cortical plate serves as an indirect multi-scale spatiotemporal biomarker until we can directly study synapse formation in the human cortex and evaluate their specific damage at the circuitry and network level. In addition, we suggest that a study of the synapse-rich compartment could be performed using macroscale imaging studies already after 12–14 weeks of gestation, i.e., after midfetal expansion of the subplate. New sophisticated MR approaches (Neumane et al. 2022) and well-established multimodal MR techniques, such as DTI, fMR, default mode activity, combined with transcriptional “cartography” (Wagstyl et al. 2024), large-scale MR metrics (Bethlehem et al. 2022), and increasing role of high-resolution ultrasound imaging showing cortical lamination (Namburete et al. 2023) which can be complemented by our spatiotemporal multiscale data on synaptogenesis, offer new vistas in the diagnostic approach to the SP synaptic compartment.

## Data Availability

The datasets generated and analyzed during the current study are not publicly available due to the ethical reasons, but are available from the corresponding author on reasonable request.

## Abbreviations

CP: Cortical plate
SP: Subplate
PSP: Presubplate
SPu: Subplate upper
SPi: Subplate inner
sSP: Superficial subplate
MZ: Marginal zone
EM: Electron microscopy
SGL: Subpial granular layer
PCW: Postconceptional weeks
ECM: Extracellular matrix
AChE: Acetylcholinesterase
MR: Magnetic resonance
IZ: Intermediate zone
SVZ: Subventricular zone
VZ: Ventricular zone
